# Exovesicular-Shh confers Imatinib resistance by upregulating Bcl2 expression in chronic myeloid leukemia with variant chromosomes

**DOI:** 10.1038/s41419-021-03542-w

**Published:** 2021-03-11

**Authors:** Anusha, Hamza Dalal, Sitalakshmi Subramanian, Snijesh V. P., Divya A. Gowda, Krishnamurthy H., Sharat Damodar, Neha Vyas

**Affiliations:** 1 Manipal Academy of Higher Education (MAHE), Manipal, 576104 India; 2 St. John’s Research Institute, St. John’s Academy of Health Sciences, Bangalore, 560034 India; 3 Mazumdar Shaw Medical Center, Narayana Health City, Bangalore, 560099 India; 4 St. John’s Medical College and Hosptial, St. John’s Academy of Health Sciences, Bangalore, 560034 India; 5 National Centre for Biological Sciences, Tata Institute of Fundamental Research, UAS-GKVK Campus, Bellary Road, Bangalore, 560065 India

**Keywords:** Cancer therapeutic resistance, Chronic myeloid leukaemia

## Abstract

Chronic myeloid leukemia (CML) patients with complex chromosomal translocations as well as non-compliant CML patients often demonstrate short-lived responses and poor outcomes on the current therapeutic regimes using Imatinib and its variants. It has been derived so far that leukemic stem cells (LSCs) are responsible for Imatinib resistance and CML progression. Sonic hedgehog (Shh) signaling has been implicated in proliferation of this Imatinib-resistant CD34(+) LSCs. Our work here identifies the molecular mechanism of Shh-mediated mutation-independent Imatinib resistance that is most relevant for treating CML-variants and non-compliant patients. Our results elucidate that while Shh can impart stemness, it also upregulates expression of anti-apoptotic protein—Bcl2. It is the upregulation of Bcl2 that is involved in conferring Imatinib resistance to the CD34(+) LSCs. Sub-toxic doses of Bcl2 inhibitor or Shh inhibitor (<<IC50), when used as adjuvants along with Imatinib, can re-sensitize Shh signaling cells to Imatinib. Our work here highlights the need to molecularly stratify CML patients and implement combinatorial therapy to overcome the current limitations and improve outcomes in CML.

## Introduction

Chronic myeloid leukemia (CML) is caused mainly due to 9:22 chromosomal translocation. The resultant BCR-ABL fusion protein codes for a constitutively active tyrosine kinase, resulting in their uncontrolled cell division. BCR-ABL(+) cells thus accumulate in the blood—a hallmark of ~90% of CMLs. Since 2001, targeted therapy using tyrosine kinase inhibitor (TKI) drugs (Imatinib and its variants) has been highly successful in eradicating the CD34(–) BCR-ABL(+) cells and has improved outcomes in CML.

Chromosomal variants of CML (CML-variants) have been reported in 5–10% of the cases. These variants contain the classical 9:22 translocation and at least one more additional breakpoint in a different chromosome(s). Response to TKIs in these CML-variants remains controversial. They initially respond to Imatinib with the same efficiency as the 9:22 CML patients in chronic phase (CML-CP)^1^, but the responses are often short lived^2–4^. These patients require bone marrow (BM) transplants eventually—an approach filled with different types of challenges including socioeconomic issues.

Non-compliance to the prescribed therapy, especially in Indian patients, is a significant issue even in CML-CP^5,6^. It is observed even after the introduction of a generic version of Imatinib. Almost one-third of Indian patients fall in the non-compliant category and fail to respond to Imatinib or its variants when re-introduced, leading to their poor survival^5,6^. Significantly, the average age of CML presentation in India is much younger, i.e., 32–42 years (compared to Europe, 55 years or America, 66 years)^7–9^. Identifying the molecular mechanism of therapy resistances to improve outcomes in CML-variants and non-compliant patients thus becomes essential.

Imatinib and its variants work efficiently on CD34(–)-BCR-ABL(+) cells. However, they fail to work on the CD34(+)-BCR-ABL(+) leukemic stem cell (LSC) population. Sonic hedgehog (Shh) signaling is implicated in the proliferation of these LSCs^10,11^. In human BM, stromal cells can act as a source for Shh^12,13^. Shh expression is also observed in leukemic cells^14,15^. Constitutively activate Shh signaling in the CML-mice via an activated-mutant Smoothened-transgene (*SmoM2*) increases LSC population, while the loss of *smoothened* (*smo–/–*) leads to depletion of these LSCs^10,11^. Inactivation of Shh signaling using Smo-null mice or via Smo inhibitor (Cyclopamine) can increase the overall survival of CML-mice^10,11^. Using primary BM aspirates from CML-mice and CML patients, it is demonstrated that the Shh inhibitor drugs are more effective in reducing long-term culture-initiating cells^16,17^. Hence, it is derived that Shh signaling promotes Imatinib resistance by regulating the proliferation of CD34(+)-LSCs^18^.

In this study using CML cell line and patient samples, we identify the molecular mechanism of Shh signaling-mediated mutation-independent therapy resistance. Our work suggests that while Shh signaling can upregulate stemness, it is the Bcl2 expression that imparts Imatinib resistance. Autocrine Shh signaling is upregulated in non-compliant patients and CML-variants. Furthermore, we find that Shh is predominantly secreted in CML patient-derived BM plasma on exovesicles for imparting Imatinib resistance. Smo inhibitor or Bcl2 inhibitor, when used as a low-dose adjuvant, can overcome this Shh-mediated therapy resistance and re-sensitize the leukemic cells to Imatinib. Our study here suggests that Shh signaling-mediated Bcl2 upregulation can be one of the major players leading to poor outcomes in non-compliant patients and CML-variants.

## Results

### Shh is produced by BM leukemic cells in CML-variants and non-compliant patients

**Fig. 1:**
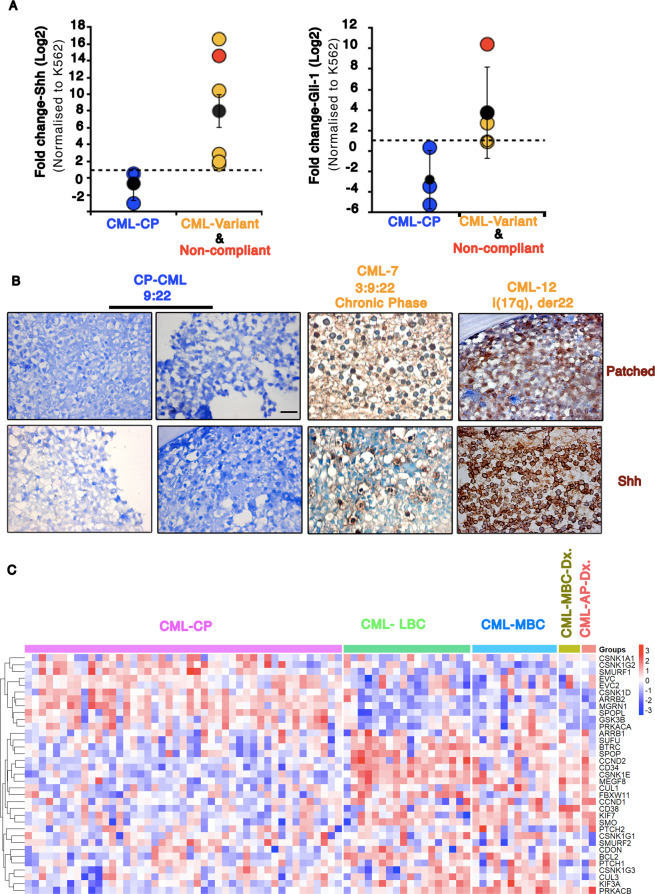
Shh expression and signaling are significantly upregulated in CML-variants and non-compliant patients. **A** Graph representing fold change in Shh and Gli1-mRNA (in log2 scale) from chronic phase CML (CML-CP) patients (*n* = 3; blue data points); chronic phase CML with variant chromosomal translocation (*n* = 5; orange) and non-compliant patients (*n* = 1; red) using RT-PCR. Reference line (black dotted line) represents the expression in normalizing control (K562 cells). *P* values, *<0.05. Statistical analysis done using *t*-test. **B** Immunohistochemistry for Shh and Patched1 using bone biopsy samples of CML patients in CML-CP (9:22; CML-3&8) and chronic phase CML with variant chromosomal translocation (CML-7, CML12). Blue, hematoxylin. Scale bar 20 μm. **C** Heatmap showing expression of Shh signaling pathway genes (as per KEGG pathway) using RNA-seq data from high-risk CML patients^1^. Patients were in Chronic Phase at diagnosis (CP Dx.) with progression to lymphoid blast crisis (LBC) or myeloid blast crisis (MBC). Three patients were in MBC at diagnosis (MBC Dx.). Two patients were in accelerated phase at diagnosis (AP Dx.) and progressed to MBC. The gene expression profiles depict the clear distinction between CML-CP from other stages.

Shh signaling has been implicated in Imatinib resistance^10,11,16,17^. To identify if Shh signaling is also upregulated in CML-variants and non-compliant Indian patients, we collected 12 CML BM aspirates. Out of 12 samples, we could identify 3 CML-CP patients, 2 non-compliant patients (9:22), and 5 CML-variants (Supplementary Table 1). We excluded two samples from the analysis due to technical issues (Supplementary Table 1). We performed RT-PCR-based analysis on BM-derived leukemic cells to evaluate Shh and Gli-1 (target gene and transcription factor) transcripts (Fig. 1A). Shh transcripts are found to be significantly higher in non-compliant and CML-variants (Fig. 1A). Gli-1 transcripts are also upregulated, but not in all samples (Fig. 1A). Here, the blast crisis patient-derived line, K562, is our reference. Hence, it is not surprising to find that in CML-CP, Shh, and Gli-1 expression are lower than our reference (black dotted line in Fig. 1A). As reported^7,9^, even in this small sample size most of the patients represent a much younger age group (Supplementary Table 1).

Furthermore, we used bone biopsy samples from these patients and validated the RT-PCR-based findings using an immunohistochemistry (IHC)-based approach. Upregulation in Shh and Patched-1 (receptor and a target gene of Shh signaling) protein levels in CML-variants is evident even here, unlike CML-CP patients (Fig. 1B). K562 and Shh-K562 served as negative and positive controls, respectively (Supplementary Fig. 1). Overall, our results here demonstrate significant upregulation of Shh expression and signaling in CML-variants and non-compliant CML patient.

To understand the expression profile of Shh signaling pathway genes in other independently reported CML studies, we analyzed the RNA-seq data from Branford et al.^19^ (80 samples) and microarray data available in the Gene Expression Omnibus data repository (GSE130404: 96 samples and GSE4170: 119 samples (www.ncbi.nlm.nih.gov/geo)^20^. Upregulation in Shh pathway genes is observed in RNA-seq data from high-risk CML patients. Here, most patients were in CML-CP at diagnosis (CML-CP Dx.). Patients who progressed to lymphoid blast crisis (LBC) or myeloid blast crisis (MBC) or once who were diagnosed in advance stages (AP Dx. or MBC Dx.) demonstrate upregulated Shh pathways components (Fig. 1C). Using microarray-based evaluation also, progressive CML patients demonstrate upregulation in Shh pathway genes (Supplementary Fig. 2). However, we could not find significant levels of Shh or Indian Hedgehog genes in either of these analyses. On the other hand, available literature on CML-variants is very sparse. We could not find any information on Shh signaling or gene expression analysis in CML-variants.

### Shh signaling can impart stemness and Imatinib resistance

**Fig. 2:**
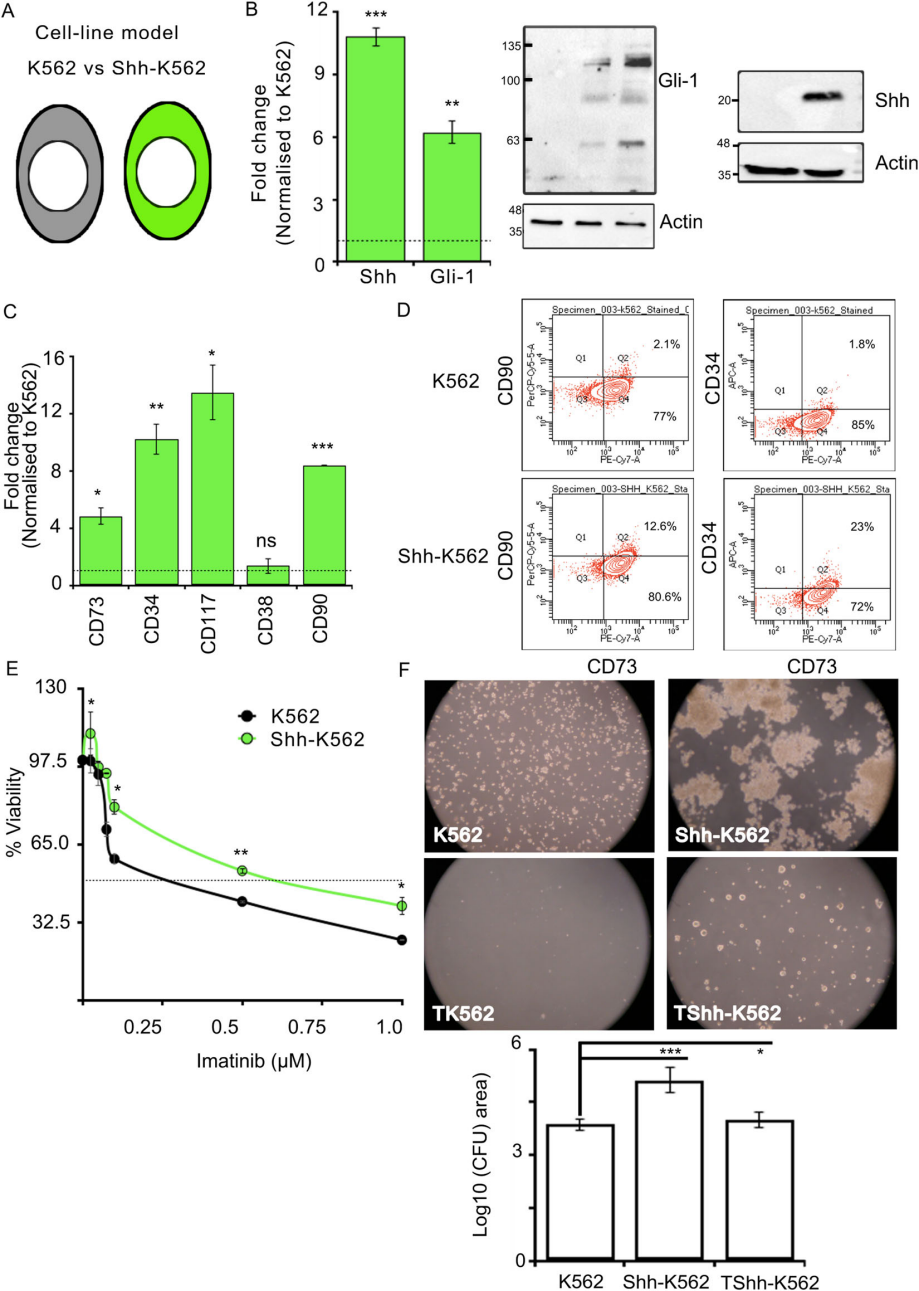
Shh signaling promotes stemness and imparts Imatinib resistance. **A** Cartoon representing cell line model used, K562 (in black) and Shh-K562 (in green). **B** Shh and Gli-1-mRNA expression in Shh-K562 determined using RT-PCR, normalized to K562. Bar represents mean ± SD from three biological repeats. Western blot demonstrating expression of Gli-1 and Shh proteins in K562 and Shh-K562. Actin is used as loading control. **C** Levels of stemness markers, CD73, CD34, CD117, and CD90, and myeloid differentiation marker (CD38) in the Shh-K562 cell line determined using RT-PCR, normalized with K562. Bar represents mean ± SD from two biological repeats. **D** Representative images of FACS-based analysis of CD90, CD34, and CD73 expression in K562 and Shh-K562. Experiment was done with two biological and technical repeats with similar results. **E** Concentration curve for Imatinib to identify IC50 at 72 h, Shh-K562 (green line) compared to K562 (control; black line graph). Line graph represents mean ± SD from three biological repeats. **F** Images demonstrating colony forming units (CFU) using K562, Imatinib-treated K562 (TK562), Shh-K562, and Imatinib-treated Shh-K562 (TShh-K562). Graph represents average area of the colonies (30–100 colonies per condition) from duplicate experiments, in Log_10_ scale. For all images, *P* values, *<0.05, **<0.01, and ***<0.001. Statistical analysis done using *t*-test.

To address the molecular mechanism of Shh-mediated Imatinib resistance, we generated a cell-based model that can recapitulate its effects in culture conditions. For this, we overexpressed Shh-cDNA in K562 cells (Fig. 2A). The upregulation of Shh expression was ascertained both at mRNA and protein levels (Fig. 2B). Shh-K562 cells can also upregulate endogenous Gli-1 mRNA and protein (Fig. 2B). These results suggest that K562 cells are Shh signaling competent and can be used to identify the molecular mechanism of Imatinib resistance.

Shh signaling is known to activate the proliferation of LSCs. We hence evaluated known LSC markers, i.e., CD34, CD117, CD90, and CD73. In Shh-K562 cells, we find upregulation of these markers via RT-PCR as well as FACS-based analysis (Fig. 2C, D). CD105, used to identify mesenchymal cell fate with CD73 and CD90, was not detectable in K562 or Shh-K562 (data not shown). Our results here support the claim so far that Shh signaling can impart stemness.

Next, we treated K562 and Shh-K562 with different concentrations of Imatinib to evaluate their responses (Fig. 2E). As reported earlier, Shh-K562 cells require higher Imatinib concentration to achieve 50% inhibitory concentration (IC50) compared to K562 cells at 72 h (Fig. 2E and Supplementary Fig. 3A). In the colony-formation assay, Imatinib-treated Shh-K562 cells were able to form visible colonies after 21 days of culturing on methylcellulose plates, unlike K562 cells (Fig. 2F). Shh-K562 demonstrates a growth advantage over K562 cells in untreated condition (Supplementary Fig. 3B and Fig. 2F). In corroboration with these findings, untreated Shh-K562 cells also have upregulated CyclinD1-levels compared to K562 cells (Supplementary Fig. 3C).

We thus confirm that Shh signaling can activate stemness, impart Imatinib resistance, and in the absence of Imatinib, it confers a growth advantage to leukemic cells.

### Shh signaling imparts Imatinib resistance by upregulating Bcl2 expression

The molecular mechanism of Shh-mediated therapy resistance was probed by analyzing the status of known players in drug resistance. We analyzed expression of a metabolic regulator (LDH), cell cycle regulators (CyclinD1, P27, P21), drug efflux pumps (ABCD1, ABCB1), autophagy regulators (Atg12, Atg9), and apoptosis regulators (Bcl2, Bax) in Imatinib-treated K562 and Shh-K562 cells.

**Fig. 3:**
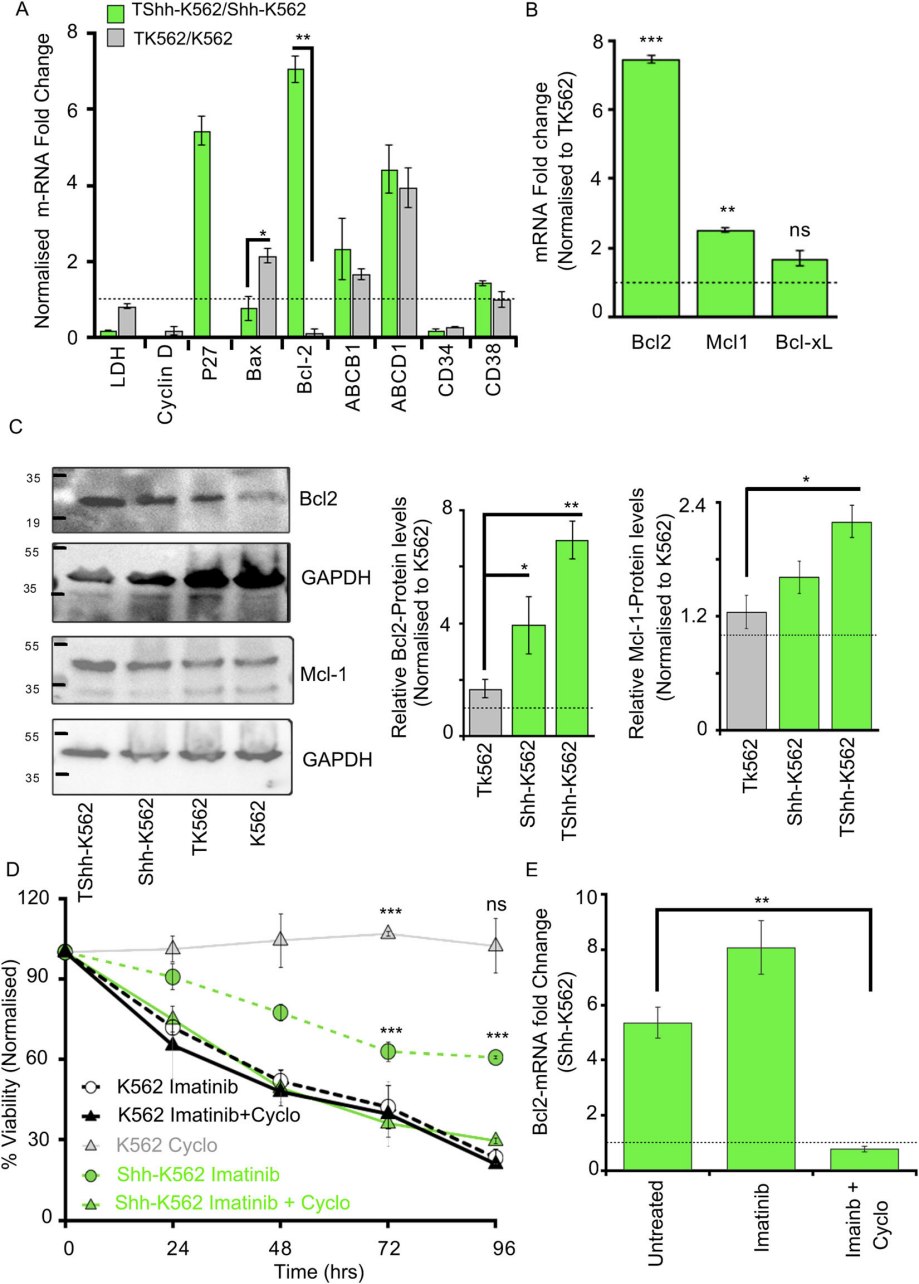
Shh signaling imparts Imatinib resistance via Bcl2 upregulation. **A** RT-PCR-based fold change determination in expression of various genes known for their role in drug resistance. Imatinib-treated Shh-K562 cells (TShh-K562) and treated K562 (TK562) normalized to their respective untreated controls, at 72 h. Bars represents mean ± SD from three biological repeats. **B** mRNA-fold change of three anti-apoptotic genes, *Bcl2*, *Mcl-1*, *Bcl-xL*, measured using RT-PCR in Imatinib-treated Shh-K562 (TShh-K562) normalized to Imatinib-treated K562 (TK562). Bars represents mean ± SD from two biological repeats. **C** Western blot representing expression of Bcl2 and Mcl-1 from untreated and Imatinib-treated cells (K562, Shh-K562, TK562, and TShh-K562). GAPDH is used as loading control. The graph represents densitometric analysis of relative protein levels normalized to untreated K562. Bar represents mean ± SD from three biological repeats. **D** Time-curve representing viability (MTT assay) of K562 (black dotted line) and Shh-K562 (green dotted line) cell lines after treatment with Imatinib (0.5 µM); K562 (black solid line) and Shh-K562 (green solid line) for Imatinib (0.5 µM) + Cyclopamine (25 nM) treated conditions; gray solid line represents viability of K562 after Cyclopamine treatment (25 nM). Graph represents mean ± SD from three biological repeats. **E** RT-PCR data determining the level of Bcl2-mRNA in K562 and Shh-K562 after treatment for 72 h with either vehicle control (untreated), Imatinib-alone, or Imatinib + Cyclopamine normalized to their respective K562 controls (black dotted line at 1). Bar represents mean ± SD from two biological repeats. For all assays, *P* values, *<0.05, **<0.01, and ***<0.001. Statistical analysis done using *t*-test.

Significant upregulation in Bcl2-mRNA and protein was observed in Shh-K562 cells in untreated and Imatinib-treated conditions (Fig. 3A–C and Supplementary Fig. 3C). Given this result, other anti-apoptotic proteins, Mcl-1 and Bcl-xL, were also evaluated (Fig. 3B, C and Supplementary Fig. 4). Mcl-1 is also modestly upregulated by Shh-K562, while Bcl-xL expression is not (Fig. 3B, C and Supplementary Fig. 4). Furthermore, P27 expression (cyclin-dependent kinase inhibitor) is upregulated in Shh-K562 upon Imatinib treatment, while it was undetectable even in Imatinib-treated K562 cells (Fig. 3A). Unlike Bcl2 and P27, levels of Bax (pro-apoptotic) and CyclinD1 (cell cycle protein) are downregulated by Imatinib-treated Shh-K562 cells (Fig. 3A). ABCB1 and ABCD1-mRNA levels suggest that these efflux pumps are upregulated by Imatinib, rather than Shh signaling (Fig. 3A), while Atg12 or Atg9-mRNAs were undetectable in K562 or Shh-K562 cells with or without Imatinib (data not shown).

While Bcl2, P27, CD34, and Gli-1 are upregulated in Shh-K562, Imatinib treatment has a differential effect on their expressions. Upon Imatinib treatment, Bcl2 and P27 transcripts are further upregulated by Shh-K562, but CD34 and Gli-1-mRNAs are relatively suppressed (compare Figs. 2B, C and 3A and Supplementary Fig. 5A). Given this disparity in the regulation of Shh targets upon Imatinib treatment, we validated Shh pathway activation by an independent assay (Supplementary Fig. 5B). Smoothened (Smo), a G-protein-coupled receptor and component of the Shh signaling pathway, is stabilized when Shh signaling is activated. Endogenous Smo levels are upregulated in Shh-K562 cells with and without Imatinib treatment compared to K562 cells confirming the active status of Shh signaling (Supplementary Fig. 5B).

Shh is known to signal via primary cilium in vertebrates^21,22^. We hence evaluated if K562 and Shh-K562 cells can generate primary cilium. For this, we co-stained cells with Acetylated tubulin (AcTub) and ADP-ribosylation factor-like GTPase protein-13B (ARL13B; Supplementary Fig. 6A). We failed to identify any primary cilium in K562 (data not shown) or Shh-K562 cells with or without Imatinib (Supplementary Fig. 6A). We further used the centriole marker, Pericentrin, and AcTub to confirm our findings. Here, we also tried serum-starvation or cultured cells in methylcellulose medium with and without Imatinib. Primary cilium was not detectable in any of these different culture conditions (Supplementary Fig. 6B). Nonetheless, several cells in different ratios demonstrate primary cilium-like structure that did not colocalize with ARL13B, nor was juxtaposed with Pericentrin (Supplementary Fig. 6). We hence conclude that Shh-K562 cells can activate Shh signaling independent of primary cilium.

Corroborating with the reported role of Shh in Imatinib resistance, we observe lower sensitivity of Shh-K562 for Imatinib using viability assay and CFU assay (Fig. 2E, F and Supplementary Fig. 3A). To evaluate if Shh-K562 are late responders or resistant to Imatinib, we performed a time-course assay. The viability of Shh-K562 or K562 was examined after treatment with 0.5 μM of Imatinib from 24 to 96 h (Fig. 3D). Imatinib was supplemented only once, at 0 h. Shh-K562 cells fail to demonstrate improved sensitivity to Imatinib even at 96 h when only 10–20% K562 cells are viable (Fig. 3D). We also evaluated the effect of different concentrations of Cyclopamine-alone and as an adjuvant with 0.5 μM Imatinib on cell-viability (Supplementary Fig. 7A–C). Shh-K562 demonstrates higher sensitivity to Cyclopamine (IC50 at 71.67 ± 12.74 nM) compared to K562 cells (IC50 at 126.67 ± 4.04 nM) (Supplementary Fig. 7A, B). We find that Cyclopamine can improve Imatinib response in Shh-K562 cells even at a sublethal dose (25 nM; Supplementary Fig. 7C). We hence performed the time-course assay with 0.5 μM Imatinib + 25 nM Cyclopamine (Fig. 3D). At this concentration, Cyclopamine does not show any toxicity on K562 cells (gray line, Fig. 3D), but the sensitivity of Shh-K562 cells to Imatinib improved (compare dotted green and solid green line graph, Fig. 3D). Bcl2 is the target of Shh signaling (Fig. 3A–C), we hence compared levels of anti-apoptotic proteins (Bcl2, Mcl-1, and Bcl-xL) with and without Cyclopamine as an adjuvant. Both Bcl2 and Mcl-1 expression were significantly downregulated in Imatinib + Cyclopamine-treated cells (Fig. 3E and Supplementary Fig. 4A), unlike Imatinib-alone or untreated control, while Bcl-xL levels remain unperturbed by the status of Shh signaling (Supplementary Fig. 4B). Our results here demonstrate that Shh signaling when inhibited re-sensitizes the leukemic cells to Imatinib by downregulating Bcl2 &/or Mcl-1 protein levels.

**Fig. 4:**
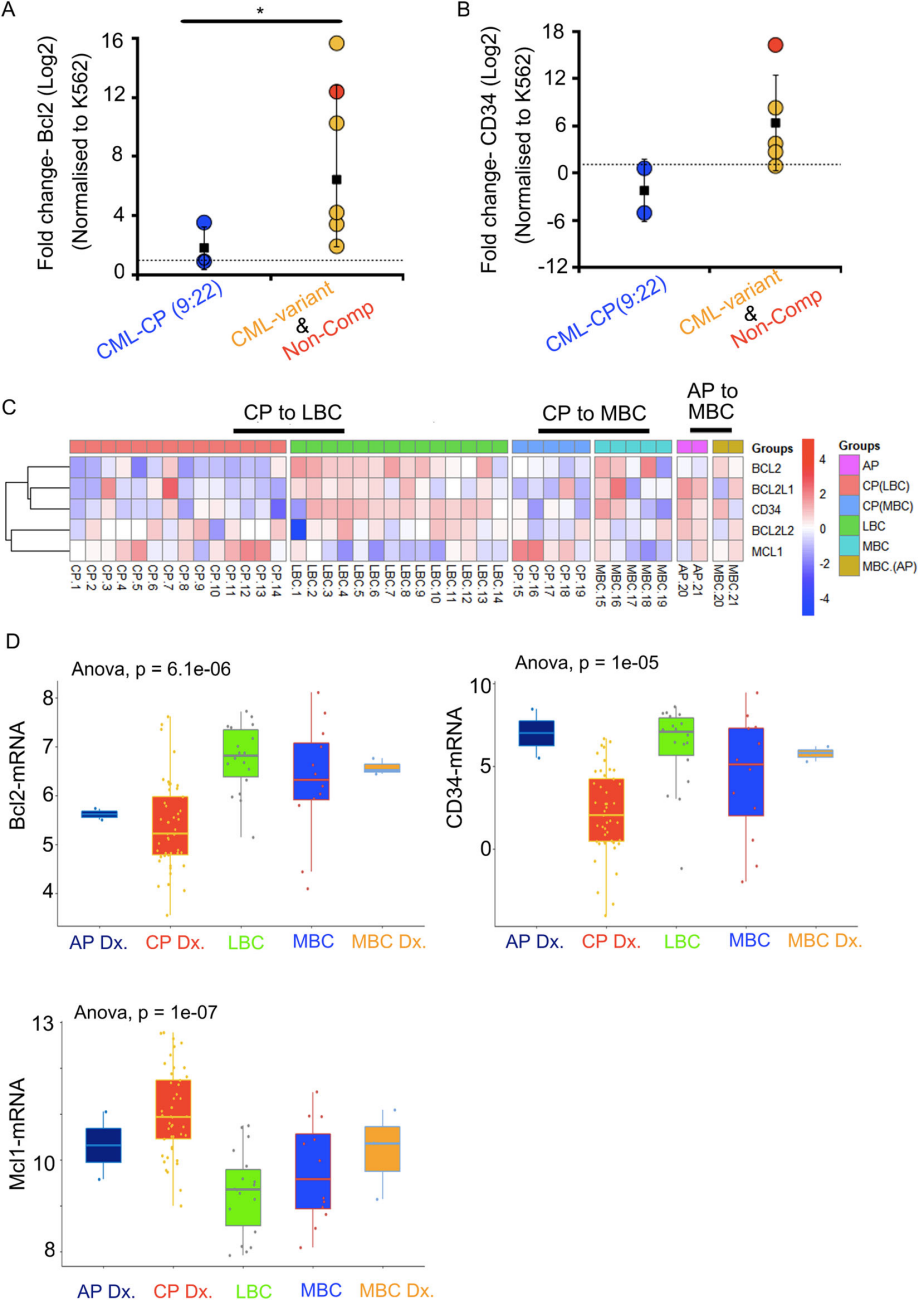
Bcl2 and CD34 are upregulated in progressive CML patients. **A** RT-PCR data representing fold change in Bcl2 and CD34 levels using BM aspirates derived from CML patient samples (*y*-axis = log2 scale). **B** Heatmap demonstrating relative expression profile of anti-apoptotic genes (Bcl2, Bcl2l1/Bcl-xL, Bcl2l2/Bcl-w) and stem cell marker (CD34) using matched CML samples. Most of the patients were in chronic phase at diagnosis (CP Dx.) with progression to lymphoid Blast crisis (LBC) or myeloid blast crisis (MBC). Two patients were in accelerated phase at diagnosis (AP Dx.) and progressed to MBC. The pattern of expression signifies the change in the trend of regulation of BCL2, BCl2L1, CD34, MCL-1 in progressive, poor responder CML patients. **C** Boxplot representing the distribution of Shh target genes BCL2, Mcl-1, and CD34 expression at various stages of the disease using data from Branford et al. **D** Significant upregulation of Bcl2 (*P* value = 6.1 × 10^–6^) and CD34 (*P* value = 1 × 10^–6^) in LBC is observed, while Mcl-1 expression is downregulated in progressive cases. Statistical analysis done using one-way ANOVA test.

As expected, Bcl2 levels are upregulated in the BM-derived leukemic cells from CML-variants and non-compliant patients (Fig. 4A). CD34 also shows the same trend but not in all patients (Fig. 4B). We then evaluated Bcl2, Mcl-1, and CD34 in the RNA-seq data from high-risk CML patients. Here again, the anti-apoptotic proteins (Bcl2, Bcl-xL(Bcl2L1), and Bcl-w(Bcl2L2)) are upregulated in progressive cases (Fig. 4C, D and Supplementary Fig. 8). Mcl-1, however, demonstrates a reverse trend (Fig. 4C, D). Interestingly, pro-apoptotic proteins are upregulated in CML-CP patients compared to progressive cases (Supplementary Fig. 8). These data from primary patient-derived cells corroborate with our findings (Fig. 4C, D).

**Fig. 5:**
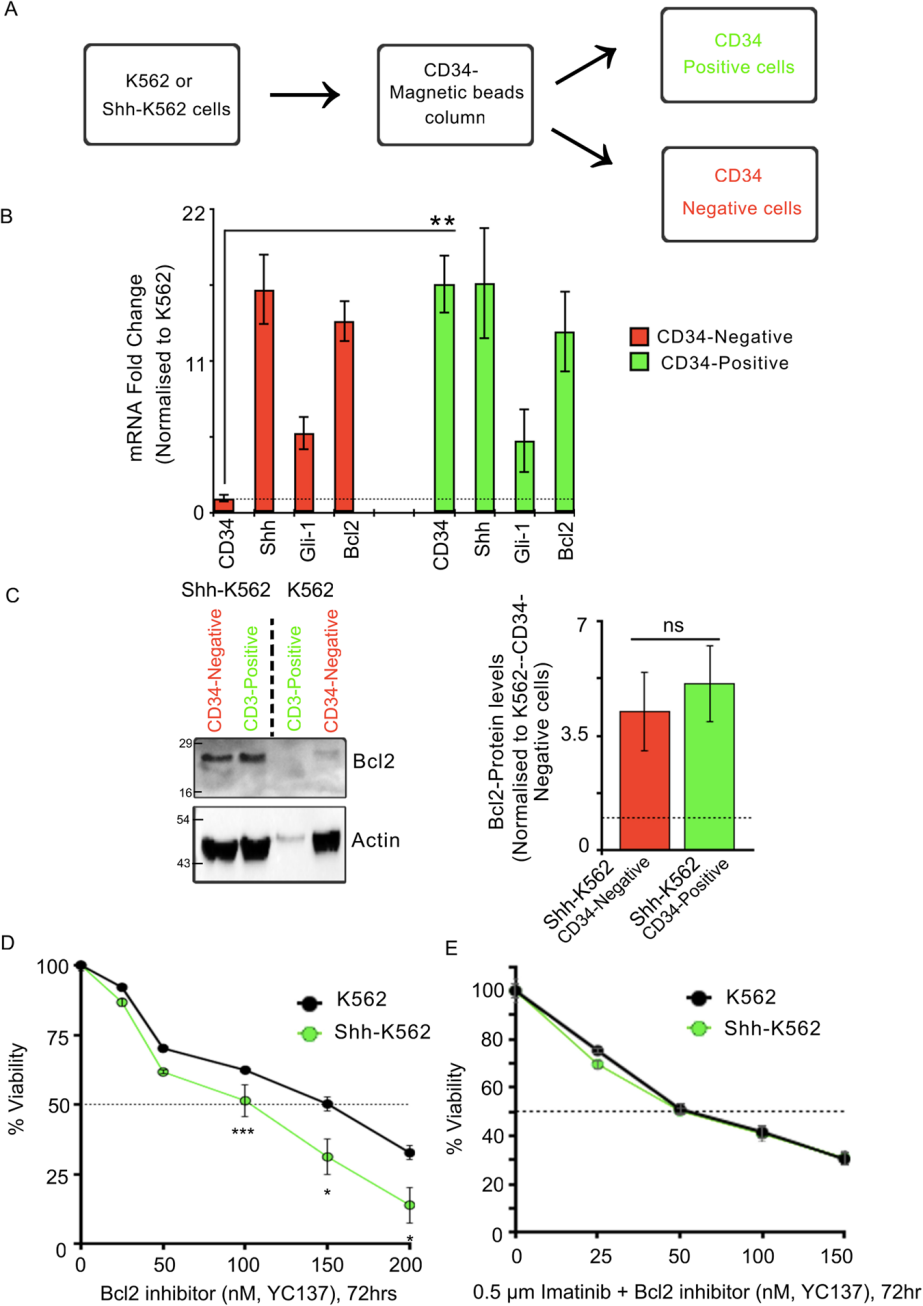
Stemness and Bcl2 are independent targets of Shh signaling. **A** Flowchart representing method for obtaining CD34(+) and CD34(–) cells from K562 and Shh-K562 cell lines. **B** Graph depicts RT-PCR-based fold change in expression levels of *CD34*, *Shh*, *Gli-1*, and *BCl2* in fractionated CD34(+) (green bars) and negative cells (red bars). *P* values derived from two biological repeats. **C** Western blot representing Bcl2 protein levels in CD34(+) and CD34(–) Shh-K562 and K562 cells. Actin used as loading control. Graph represents densitometric analysis of relative Bcl2 levels in Shh-K562-CD34(+) (red bar) and Shh-K562-CD34(–) (green bar) cells using K562-CD34(–) cells as normalizing control. Bar represents mean ± SD from two biological repeats. **D** Viability of Shh-K562 (in green) and K562 cells (in black; control) after treatment with different concentration of YC137 (Bcl2 inhibitor II) to identify IC50 at 72 h. Untreated cells at respective time-point used as reference. Line graph represents mean ± SD from three biological repeats. **E** Percentage viability of Shh-K562 (in green) and K562 cells (in black; control) after treatment with Imatinib (0.5 μM) + different concentration of YC137 (Bcl2 inhibitor II) to identify lowest effective concentration. Untreated cells at respective time-point used as reference. Line graph represents mean ± SD from three different experiments. *P* values, *<0.05, **<0.01, and ***<0.001. Statistical analysis done using *t*-test.

LSCs and Shh signaling are implicated in Imatinib resistance. Given the differential regulation of CD34 and Bcl2 transcripts in presence of Imatinib, we wanted to evaluate if the expression of these proteins is independent of each other. For this, we fractionated the CD34(+) cells from CD34(–) cells using CD34-conjugated magnetic beads (Fig. 5A). As expected, we could isolate both these fractions using Shh-K562 but were unable to find significant CD34(+) cells using K562. We then evaluated the expression of Bcl2, Shh, and Gli-1-mRNA using CD34(–) and CD34(+) fractions. The reliable fractionation of these pools was confirmed by looking at CD34 transcripts (Fig. 5B). Both these fractions have equal levels of Shh and Gli-1 transcript, suggesting the activated status of Shh signaling. Bcl2-mRNA and protein levels were upregulated both in CD34(+) and CD34(–) fractions (Fig. 5B). We next performed a drug sensitivity assay using different concentration of YC137, a specific inhibitor of Bcl2^23^ (Fig. 5D). Like Cyclopamine, Bcl2 inhibitor also shows higher sensitivity in Shh-K562 cells compared to K562 cells (Fig. 5D and Supplementary Fig. 7A). Once again, sublethal levels of YC137, when used in combination with Imatinib, could re-sensitize Shh-K562 cells to Imatinib (Fig. 5E). Thus, while Shh signaling can upregulate stemness, it is Bcl2 upregulation that imparts Imatinib resistance.

### Shh is secreted predominantly on exovesicles for imparting Imatinib resistance

**Fig. 6:**
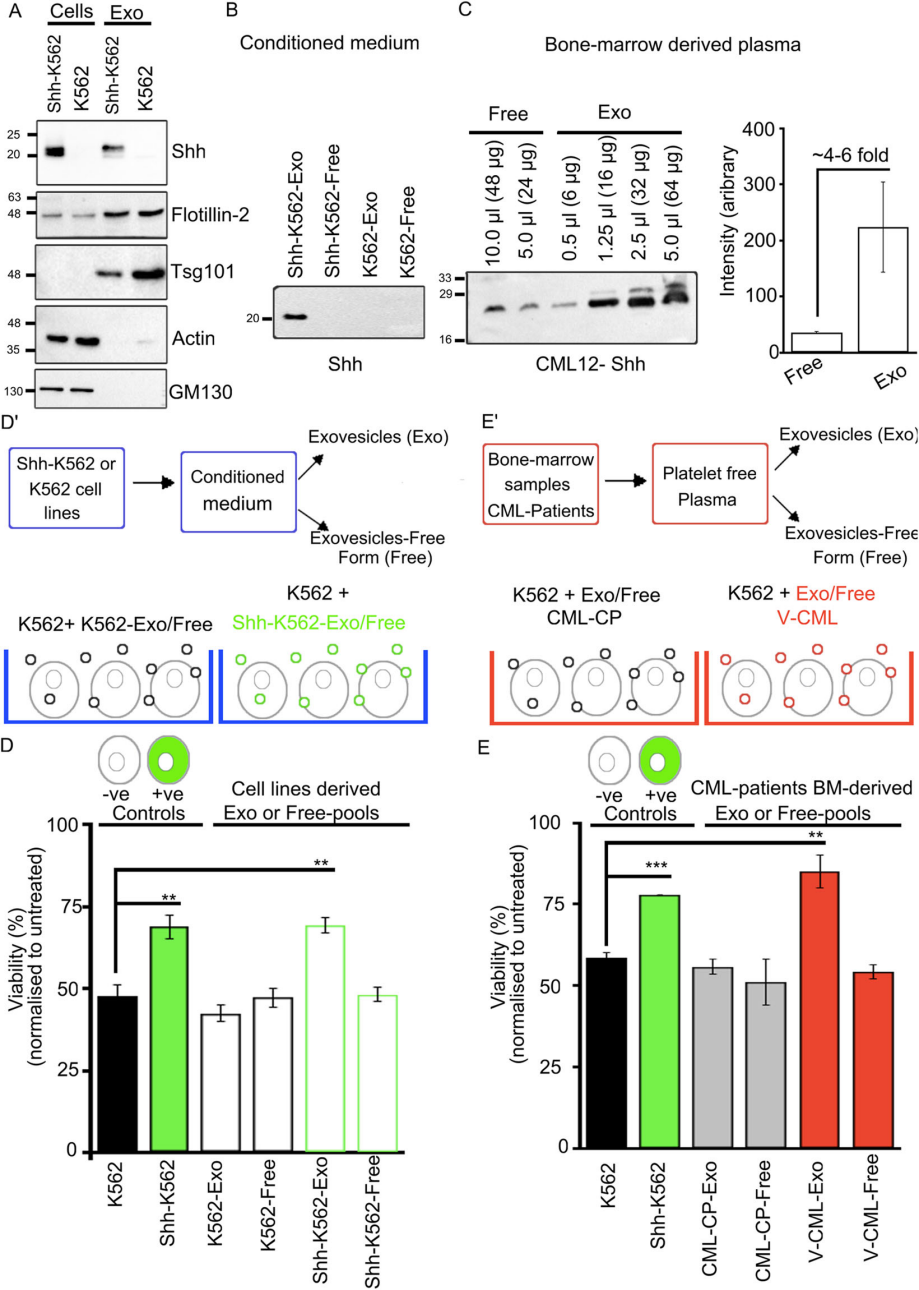
Exovesicular-Shh imparts Imatinib resistance. **A** Western blot-based analysis of the cell lysate and Exo-fraction isolated from Shh-K562 and K562. Shh, Actin (cytosolic protein), GM130 (a golgi marker), Tsg101, and Flotilin2 (exovesicle markers) analyzed. Data from two to three biological repeats each. **B** Western blot evaluating relative levels of Shh in Exo and free-fractions derived from Shh-K562 and K562 condition media. Equal total protein loaded in each well for analysis (*n* = 4). **C** Western blot representing presence of Shh in extracellular fractions Exo and free-fractions derived from BM sample from CML patient (CML12, CML-variant). Different concentrations of total protein were loaded per well to evaluate relative enrichment of Shh in each fraction, as indicated. Graph represents densitometric estimation of relative protein levels normalized to total amount of protein loaded per well. **D’** Schematic represents the assay procedure using conditioned medium. **D** Bar graph represents viability of K562 cells pretreated with Exo-fraction or free-fraction derived from K562 or Shh-K562 (12-h pre-treatment), followed by treatment with 0.5 µM Imatinib for 72 h. Imatinib-treated K562 cells (black bar) and Shh-K562 cells (green bar) used as negative and positive controls, respectively. Bar represents mean ± SD from three different experiments. **E’** Schematic represents the assay procedure using BM plasma. **E** Bar graph represents viability of K562 cells after 12-hr pre-treatment with Exo or free-fraction derived from CML patients BM (red bar, V-CML; CML-4, 9, 12) (*n* = 3); gray bar, CML-CP (9:22; CML-1, 3, 8) (*n* = 3), followed by treatment with 0.5 µM Imatinib for 72 h. Imatinib-treated K562 cells (black bar) and Shh-K562 cells (green bar) used as negative and positive controls, respectively. Data represent mean ± SD from three different CML patient-derived fractions. *P* values, *<0.05, **<0.01, and ***<0.001. Statistical analysis done using *t*-test.

Shh is secreted extracellularly in vesicular and non-vesicular forms. These forms are likely to have differential signaling abilities but remain unexplored in disease conditions^24,25^. To evaluate this, we isolated exovesicles (Exo-fraction) using conditioned media derived from K562 and Shh-K562 cells. Shh is predominantly detected in exo-fractions, along with markers such as Tsg101 and Flotillin-2 (Fig. 6A). Conditioned medium after exovesicles isolation is also collected to evaluate the presence of non-vesicular or free form of Shh (free-fraction), their relative levels, and role in Imatinib resistance. No detectable Shh was identified in free-fractions or in K562-conditioned medium (Fig. 6B). Exo-fractions did not show detectable amounts of Actin or Golgi maker—GM130 (Fig. 6A). This suggests that the vesicles isolated from the conditioned medium are enriched for the secreted exovesicles, ruling out any non-specific contribution due to cell lysis or cell death.

We also isolated Exo and free-fractions using patient-derived BM plasma. Relative Shh levels in Exo-fractions and free-fractions were evaluated using equal total protein (30 μg) and densitometric analysis (Fig. 6C and Supplementary Fig. 9A). Shh is secreted predominantly on exovesicles in BM of most patients (Fig. 6C and Supplementary Fig. 9A); rarely patients with higher levels (CML11) or equal levels (CML10) of free form of Shh were also identified (Supplementary Fig. 9A, B). To our surprise, detectable Shh was also present in plasma-derived exovesicles from CML-CP patients (CML-1, 3; Supplementary Fig. 9A), though Shh expression or signaling was not detectable in leukemic cells (Fig. 1A, B).

We used these fractions to determine their ability to activate Shh signaling and impart Imatinib resistance (Fig. 6D’, E). For this, K562 cells were pre-incubated with respective Exo or free-fractions for 12 h and then treated with 0.5 μM Imatinib for 72 h. Equal total protein (50 μg) was used from each fraction. K562 cells were used as negative (black bars, Fig. 6D, E) and Shh-K562 cells as positive control (green bars, Fig. 6D, E). Incubation with either Shh-K562-Exo or Exo-fraction derived from CML-variant or non-compliant patients (V-CML) (but not Shh-K562-Free, K562-Exo/Free-fractions) could impart similar protection to K562 cells, as seen for Shh-K562 cells (Fig. 6D, E). Exo-fraction from CML-CP patients demonstrates viability similar to K562 (gray bars, Fig. 6E). Our results here demonstrate the role of exovesicular-Shh derived from Shh-K562, CML-variants, or non-compliant patients’ BM plasma in imparting Imatinib resistance. It also indicates that the concentration of Shh in CML-variants or non-compliant patients’ plasma is higher compared to CML-CP patients.

### Sublethal levels of Shh inhibitor or Bcl2 inhibitor can be used as adjuvants to re-sensitize leukemic cells to Imatinib

**Fig. 7:**
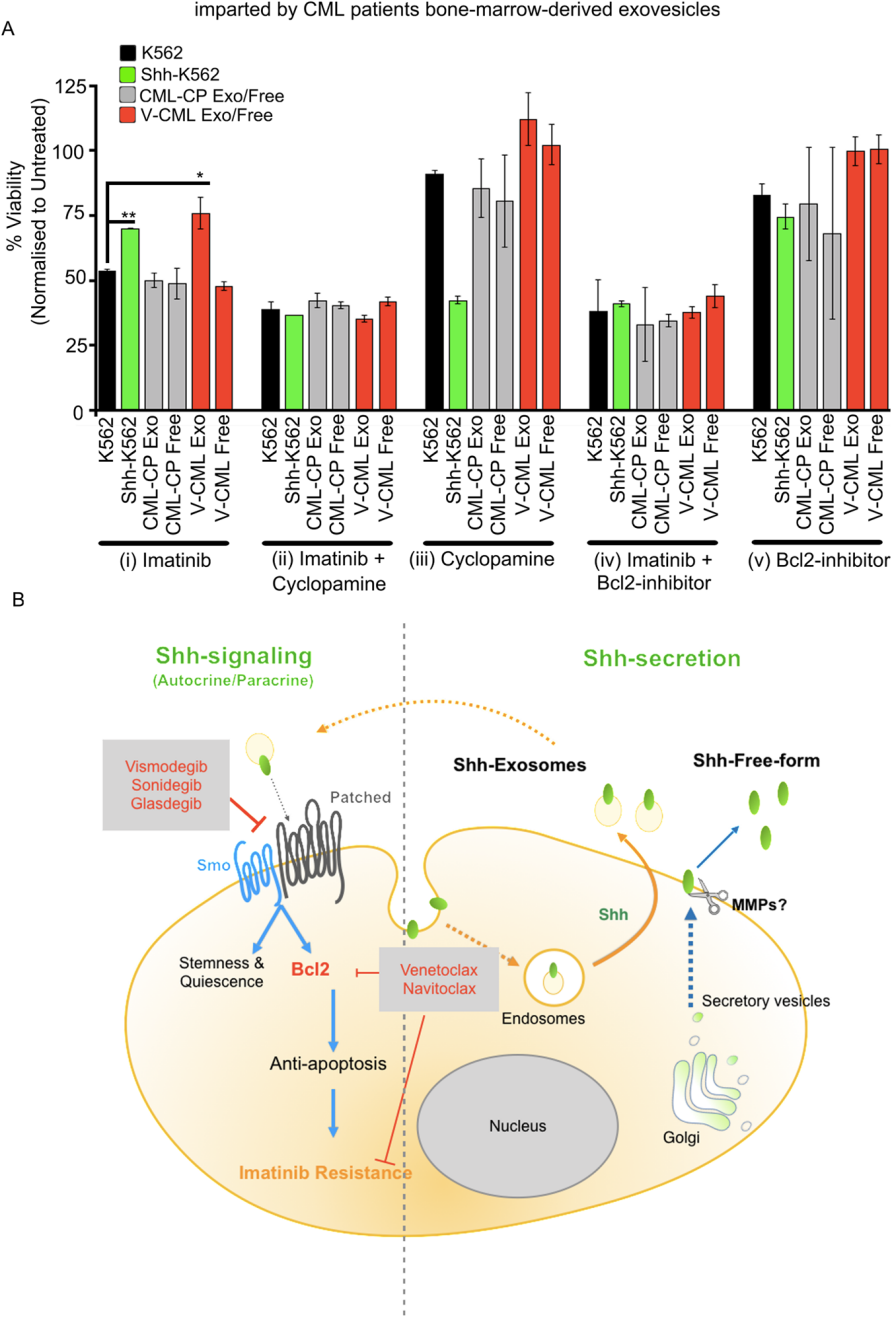
Shh inhibitor or Bcl2 inhibitor can be used as adjuvants to overcome Imatinib resistance imparted by CML patient’s BM-derived exovesicles. **A** Viability of K562 cell line treated with Exo or free-fractions derived from CML-CP patients (CML-1 or CML-8) or V-CML (CML-4 or CML-9) for 12 h, followed by treatment with 0.5 µM Imatinib (**i**), 25 nM Cyclopamine (**iii**), 50 nM Bcl2 inhibitor (**v**), or combined treatment (0.5 µM Imatinib + 25 nM Cyclopamine (**ii**) or 0.5 µM Imatinib + 50 nM Bcl2 inhibitor (**iv**)) for 72 h. Data represent mean ± SD from two different CML patient-derived fractions. *P* values, *<0.05 and **<0.01. Statistical analysis done using *t*-test. **B** Cartoon representing mechanism of secretion of different extracellular forms of Shh and Shh-mediated Imatinib resistance in leukemic cells. Available FDA-approved drugs indicated in red text, which can be used to overcome Shh-mediated therapy resistance.

We then evaluate if Cyclopamine or Bcl2 inhibitor (YC137) can be used to overcome Imatinib resistance. For this, K562 cells were treated with BM plasma-derived Exo or free-fractions from CML-variants, non-compliant (V-CML, red bars, Fig. 7A), or CML-CP patients (gray bars, Fig. 7A) for 12 h. These pretreated cells were then treated with, Imatinib + Cyclopamine, Imatinib + YC137, or single agents (Imatinib, Cyclopamine, or YC137) for 72 h (Fig. 7A and Supplementary Fig. 9C). K562 (black bars, Fig. 7A) and Shh-K562 (green bars, Fig. 7A) were used as negative and positive controls, respectively. Imatinib resistance imparted by Exo-fraction (red bars, Fig. 7A(i)) can be circumvented by using sublethal doses of Cyclopamine or Bcl2 inhibitor in combination with Imatinib (red bars, Fig. 7A(ii), (iv)). When used as single agents, these inhibitors are not effective (red bars, Fig. 7A(iii), (v)). They also do not show any significant toxicity on K562 cells (black bars, Fig. 7A(iii), (v)).

In CML11, Shh is predominantly secreted in free-fraction (~40–50 times higher than the Exo-fraction; compare Fig. 6C and Supplementary Fig. 9B). Though Shh levels are significantly higher in free-fraction from CML11 patient, it provides similar Imatinib resistance that can also be resolved using Bcl2 or Cyclopamine as adjuvants with Imatinib (Supplementary Fig. 9B, C). We also noticed that CML11 patient plasma contains a much higher total protein than other CML patients (Supplementary Fig. 10). The reason for this observed difference in the extracellular form of Shh or total protein content remains unknown.

Our results suggest that Shh is predominantly secreted on exovesicles in CML patients’ BM plasma to impart Imatinib resistance. Shh-mediated resistance to Imatinib is conferred, via the upregulation of the Bcl2 rather than via stemness (Fig. 7B). In CML-variants and non-compliant patients, Shh signaling can play a major role in contributing toward the poor outcome via Bcl2 upregulation. Expression of Shh and/or Bcl2 can be used as markers to identify non-compliant patients and CML-variants who are likely to demonstrate this mutation-independent Imatinib resistance. We envisage that identifying such patients and treating them with the proposed combinatorial therapy might help in further improving outcomes in CML. However, given the smaller sample size in our study here, this proposition needs to be further clinically tested in larger cohort(s) to evaluate the magnitude of its clinical implication.

## Discussion

The discovery of Imatinib has made a significant difference in the outcome of CML patients. Imatinib is used for almost a decade in India as a frontline monotherapeutic agent. It can maintain 80–90% of patients in the most responsive, CP for a long time. However, 10–20% of patients develop the more aggressive disease. In the current therapeutic regime, CML patients need to take the therapy for a long time (~8–10 years) to achieve deep molecular remission. This need has created additional challenges of non-compliance and drug toxicity. Often, Imatinib toxicity develops after 2 years of treatment^26^.

We find that CML-variants (in CP), despite the different additional chromosomal involvement (Supplementary Table 1), demonstrate upregulation in Shh expression and signaling compared to CML-CP patients. A non-compliant patient also demonstrates significant upregulation in Shh expression. This can be detected using RT-PCR-based approach and in IHC using the bone biopsy samples. What triggers the expression of Shh in leukemic cells from CML-variants or non-compliant (9:22) patients remains unanswered here.

The Shh-K562 stable line used here shows 8–12-fold upregulation in Shh expression. This is well within the observed Shh levels in these patients (Fig. 1A) for a reliable correlation. Using this stable line, we find that Shh expression does promote cell proliferation and imparts stemness and Imatinib resistance. However, upon Imatinib treatment, Shh rather upregulates quiescence markers P27 (Fig. 3A), and P21 (data not shown), while it downregulates CyclinD1, CD34, and Gli-1 levels. How does Shh signaling differentially regulate these targets? What role do TKIs play in the regulation of Shh target genes? These questions remain unanswered and need further investigation.

Unlike most vertebrate cells, Shh-K562 cells fail to demonstrate a distinct primary cilium (Supplementary Fig. 6). We deliberate that the presence of primary cilium-like structures could rather represent post-mitotic midbody. Midbodies are retracted in most cells post mitosis via phagocytosis but can be retained in some post-mitotic cells for functions other than cytokinesis^27,28^. Primary cilium is required for Shh signaling but there are also exceptions^29–32^. In addition, ciliary protein, ARL13B, can function outside of cilium in regulating the Shh pathway directly^33^. Such exceptions indicate that cilium can be dispensable for Shh signaling even for vertebrates, in a context-specific manner.

Preclinical studies using Shh signaling inhibitors suggest that their long-term use can induce teratogenicity, embryotoxicity, and fetotoxicity (FDA approval number: 203388). The side effects in adult animals (dogs and/or rats) include muscle spasm, impaired fertility, tremors, myoclonic jerking, or ataxia, teeth, and bone toxicity (FDA approval number: 203388, ref: 3075878). These side effects are reversible upon drug withdrawal. Side effects of Imatinib include musculoskeletal pain, muscular cramps, a decrease in bone-mineral density, and bone health^26^. Hepatic and renal damages are observed in acute cases. TKI toxicities are also reversible upon drug withdrawal. However, our results suggest that pre-mature interruption of the TKI therapy could lead to the CML progression by promoting proliferation of Shh signaling cells. These re-populated BM cells are likely to demonstrate a significant mutation-independent resistance to Imatinib and its variants via anti-apoptotic proteins (mainly Bcl2), leading to a more aggressive disease.

The use of anti-apoptotic inhibitors in blast crisis patients with upregulated CD34 levels has been proposed but the regulator of anti-apoptosis was not identified^34^. Shh signaling has been implicated in Imatinib resistance by promoting stemness^11,17,35^. Quiescent LSCs are also implicated in CML progression^36,37^. Shh signaling is a known regulator of Bcl2 expression directly via Gli-1 in normal and cancerous cells^38–40^. The use of Bcl2 inhibitor drugs is advocated in different cancers including leukemia^41,42^. Upregulation in Bcl2 and other anti-apoptotic proteins is also observed, in patients who have progressed to LBC or MBC stages (Fig. 4; 21 matched samples). Our work here can link these different observations. We now clarify that Shh itself can promote CD34(+) cell fate and quiescence. These properties might play a major role in masking the identification of Shh signaling cells when on TKI therapy but not in imparting Imatinib resistance. Shh-mediated Imatinib resistance is rather due to the upregulated expression of apoptosis inhibitor—Bcl2 (Fig. 7B).

Our study highlights the need to introduce Shh signaling and/or Bcl2 as markers to further stratify CML patients. Importantly, in the presence of Imatinib, Gli-1 and CD34 expression are relatively suppressed (compare fold change between Figs. 2B, C and 3A and Supplementary Fig. 5A), and Shh may demonstrate paracrine or autocrine signaling. We hence anticipate that Bcl2 or Smo levels might be more reliable as markers for patients who are on Imatinib treatment.

We envisage that the combinatorial therapies proposed here can prove to be effective not only in non-compliant patients but also for CML-variants in the CP to improve outcomes. Our data demonstrate that the proposed adjuvants are effective even at lower concentrations (<<IC50) and hence may prove to be more endurable for long-term use both biologically and socioeconomically. If used as adjuvants, these drugs can re-sensitize the leukemic cells to Imatinib to further improve outcomes. However, this proposition needs to be tested using primary cells and in vivo models. Systemic use of this proposed combination of drugs also requires further studies to identify any novel toxicity or side effects. In addition, evaluating the model proposed here clinically in larger cohort(s) will allow us to resolve outliers or subgroups (based on the type of chromosomal variant or otherwise) that might need other molecular considerations.

Furthermore, in culture conditions, an in vivo Shh is present predominantly on exovesicles. In one patient, Shh is predominantly secreted in free form. This patient’s plasma also demonstrated a significantly higher total protein content (Supplementary Fig. 10). This can be ascribed to the increased activity of metalloproteases^43^. MMP-9 has been implicated in generating signaling competent extracellular, non-vesicular pool of Shh^44–46^. Our results suggest that Shh present in either of these fractions can impart Imatinib resistance. However, the signaling efficiency of these extracellular forms needs a closer examination.

Detectable levels of Shh exovesicles present in the BM plasma of CML-CP patients raise several questions. What is the source of Shh in CML-CP? Does it play a role in the extended requirement of Imatinib therapy? The current requirement for maintaining CML patients on long-term TKI therapy (8–10 years) incurs a constant cost that has been unsustainable even for developed countries^47^. It might be worth evaluating if even in the Imatinib responsive CML-CP these proposed combinatorial therapies could reduce or eliminate minimal residual disease and provide a deep molecular response at a much faster rate. Our work here highlights the need to introduce Shh and Bcl2 as markers to further identify and stratify CML patients who are likely to show an inferior response on Imatinib—an approach missing in the clinics currently.

## Methods and materials

### BM sample processing

The study was approved by the institute review board (IRB Min. No. NHH/AEC-CL-2017-172, dated January 9, 2018). BM aspirates from CML patients were collected in EDTA tubes. Plasma (upper layer) was separated from BM aspirates by centrifuging at 2500 rpm for 5 min. The remaining sample was then treated with 1X RBC Lysis Buffer, 20 min on Rocker. Cells were isolated by centrifugation at 1000 rpm for 15 min. Cells were counted and divided in different tubes for RNA Extraction using TRIzol Reagent.

### Bioinformatics-based expression data processing

Normalized fragment counts (log2 counts per million) were taken from the analysis performed by Branford et al.^19^. The datasets are deposited at European Genome-phenome Archive under accession EGAS0000100307126. The dataset comprised of 80 RNA-seq samples at different stages of the CML namely AP.Dx (2 samples), CP.Dx (45 samples), LBC (18 samples), MBC (12 samples), and MBC.Dx (3 samples). The data were categorized based on the patients who had progression from a stage to another stage. We got 14 patients from CP.Dx to LBC, 5 patients from CP.Dx to MBC, and 2 patients of AP.Dx to MBC. The genes involved in Hedgehog pathway were retrieved from Kyoto Encyclopedia of Genes and Genomes (KEGG) database with accession hsa04340. The pro- and anti-apoptotic genes of BCL2 family were considered for analysis. Furthermore, the genes from normalized expression matrix were intersected with these genes to understand their role in CML. The heatmap were developed using pheatmap r-package and statistical analysis of one-way ANOVA test for multiple groups were performed using ggpubr.

The raw expression signals associated with in CML with series identifier GSE4170 and GSE13204 were downloaded from Gene Expression Omnibus (www.ncbi.nlm.nih.gov/geo)^20^. The samples selected from GSE4170 comprised of chronic phase (CP), accelerated phase (AP), and blast crisis (BP)^48^. The series GSE13204 consisted of samples from control (73 specimens) and CML (66 specimens). The detailed information about the samples are given in the Supplementary file (Supplementary Tables 2 and 3). The genes involved in the Hedgehog pathway were retrieved from KEGG database (www.genome.jp/kegg/pathway/hsa/hsa04340.html)^49^. Preprocessing of expression data was performed using R packages. To standardize and reduce the technical noise in the sample data, raw intensity signals were processed using Affy and limma packages and the raw signal values of each sample set were standardized to a median of all samples using the Robust Multiarray Average algorithm by baseline^50–52^. This algorithm normalizes the raw signals by generating a matrix of expression from the data with context correction and log2 conversion followed by quantile normalization. To understand the expression pattern, the hedgehog pathway genes retrieved from KEGG were mapped to the normalized expression data.

### MTT assay

In all, 1000–2000 cells were plated per well in a 96-well plate and incubated with the appropriate drug or without the drug as specified for the desired time. For drug sensitivity assay, BM-derived exosome-fraction or free-fraction (50 µg), isolated from plasma with the help of ExoEnrich kit, was added to cells after 12 h. Cells were then treated with desired concentrations of drugs or untreated as mentioned and cultured for 72 h. Then, 5 mg/ml MTT (10 μl) was added to the cells, 3–4 h at 37 °C. The cells were incubated with DMSO, at room temperature for 10–15 min for lysis and until purple crystals dissolve. Absorbance was measured at 540 nm within 30 min using Spectrophotometer SN 1510-01922C (Thermo Fisher Scientific), Imatinib Mesylate (Sigma-Aldrich, SML1027), Cyclopamine (Sigma 24724439), and Bcl2 inhibitor II YC137 (Sigma-Aldrich, CAS810659-53-1).

### Exovesicle isolation

Exovesicles were isolated using pre-cleaned culture media (500 μl) or BM aspirate-derived plasma (500 μl) using ExoEnrich kit (ExoCan Healthcare Technologies Pvt Ltd, India). Pellet and supernatant were obtained after processing as per manufacturer’s instructions. The isolated pellet contained exovesicles (Exo-fraction), which was resuspended in 500 μl of 1X PBS and the supernatant (~500 μl) was collected as exovesicle-free-fraction (free-fraction). Protein content of the fractions was derived using Bradford method.

### CFU assay

In all, 1000 cells (K562 or Shh-K562) were plated and cultured with or without (0.5 μM) Imatinib for 72 h in 96-well plates. Cells were then washed with PBS to remove Imatinib and plated on 1% agarose solid media along with 1.5 ml StemMACS(TM) HSC-CFU (Miltenyi Biotec) in 6-well plate for 21 days. The cells were then imaged at 10× using CKX41 Olympus microscope using Spectral Imaging camera (Model: GEA-1000A).

### FACS analysis

Unstained beads (negative beads and positive beads), single antibody, and negative beads + positive beads were used for compensation. K562 and Shh-K562 cells were harvested, stained with antibody mix, and incubated for 15 min at room temperature. Cells were then centrifuged, washed with 1X PBS, centrifuged at 1000 rpm for 5 min and fixed using 4% PFA for FACS analysis using BD LSR Fortessa X20 Analyzer. CD73 PE-Cyanine7 (mouse, Thermo Fisher Scientific), CD90 PerCP-Cyanine5.5 (mouse, Thermo Fisher Scientific), CD34-APC (mouse, Thermo Fisher Scientific), and CD105-SB436 (mouse, Thermo Fisher Scientific).

### Cell culture

K562 cell line was a kind gift from Dr. Sudhir Krishna’s lab, Bangalore, India. Shh-K562 cell line was made by transfecting Shh-Np containing pCDNA 3.1 plasmid using lipofectamine Reagent (ThermoFischer Scientific). The cells were cultured in RPMI-1640 medium (Gibco, Life Technologies) supplemented with 10% fetal bovine serum (Gibco, Life Technologies) with the addition of 1% Pen-strep (Gibco, Life Technologies) and maintained in a 5% CO_2_ humidified Galaxy 170 R incubator (Eppendorf) at 37 °C.

### RNA isolation and cDNA synthesis

RNA was extracted using the TRIzol reagent (Invitrogen). Samples were treated with chloroform (20%), vortexed briefly, and incubated at room temperature for 15 min. Samples were then centrifuged at 14,000 rpm at 4 °C for 15 min. The aqueous phase was moved to a new tube and an equal volume of isopropanol was added. Samples were incubated at room temperature for 10 min, followed by centrifugation at 14,000 rpm at 4 °C for 10 min. Pellets were washed in 95% ethanol, dried and resuspended in diethyl pyrocarbonate-treated double-distilled water. cDNA was synthesized using the Applied Biosystems High Capacity cDNA Reverse Transcription Kit according to the manufacturer’s instructions.

### RT-PCR

A total of 2 µg total RNA was used for cDNA synthesis followed by real-time PCR using Applied Biosystems SYBR Green PCR Master Mix. Real-time PCR was performed using 7500 Fast ABI Machine. Fold change in relative expression of each target gene compared to the loading control calculated using the 2(–ΔΔCt) method. A change in expression of twofold was considered significant. The primers used in qPCR are mentioned in Supplementary Table 4. GAPDH was used as an internal control.

### Western blot

Whole protein extracted from K562 cells and Shh-K562 cells were lysed using cell lysis Radio immunoprecipitation assay buffer (300 mM NaCl, 50 mM Tris, 0.5% Triton X-100 and 0.1% SDS, pH 7.4). Protein samples were denatured and separated on 14% sodium dodecyl sulfate polyacrylamide gel electrophoresis and transferred to nitrocellulose membranes. Membrane was then incubated in 5% skim Milk for 1 h at room temperature and then with primary antibodies (given in Supplementary Table 2) diluted in 5% Skim milk at 4 °C overnight. After washes, blots were incubated with anti-mouse-HRP or anti-rabbit-HRP IgG (Jackson ImmunoResearch; 1:1000) secondary antibodies at 4 °C overnight. It was used for detection blot developed using Super Signal West Pico Plus Chemiluminescent Substrate (Thermo Scientific) as per manufacturer instructions. Anti-Shh (1:500, Rabbit, CST), anti-Bcl2 (1:500, Mouse R& D systems), anti-Gli1 (1:500, Rabbit, Sigma), anti-actin (1:1000, Rabbit, Abcam), anti-GM130 (1:1000, Rabbit, Abcam), anti-Flotillin2 (1:500, Rabbit, Sigma), Tsg101 (1:500, Rabbit, Sigma).

### Immunohistochemical identification

K562 and Shh-K562 cells were fixed using 4% PFA for 15 min at room temperature. Smear was made on the charged slide and was air dried. Permeabilization was done using 0.1% Triton X-100 for 10 min at room temperature. Endogenous peroxidase activity was blocked with 3% hydrogen peroxide (H_2_O_2_) for 30 min at room temperature. After washing, antigen retrieval was done by dipping the slides in EDTA buffer (0.1 M, pH 9.0) for 15 min in water bath at 70 °C. Slides were cooled at −20 °C and were washed twice with TBST for 3 min. Slides were then put for blocking in 3% BSA for 30 min. Primary antibodies, anti-Shh (CST, Rb, 1:100) or anti-Patched (Abcam, Rb, 1:100), were added and the slides were incubated at 4 °C overnight, followed by staining with anti-rabbit HRP-conjugated secondary antibody (Jackson ImmunoResearch; 1:500) for 1 h at room temperature. Then, 2% DAB (Thermo Fisher Scientific) was added on the slides and kept until color was developed in control slides and then washed with distilled water. After that, 5% hematoxylin was added to the slides for counter staining for 3 min. The slides were then washed with distilled water, air dried, mounted using DPX (Merck), and imaged using BX51 Olympus Upright Microscope at 40× magnification.

List of antibodies used:

**Table:** 

No.	Antibody (western)	Catalog number	Company	Use
1.	Shh-rabbit	2207	Cell Signaling Technologies (CST)	Western blot (WB) and immunohistochemistry (IHC)
2.	Gli1-rabbit	SAB2700185	Sigma	WB
3.	Bcl2-mouse	ab692	abcam	WB
4.	GM130-rabbit	ab187514	abcam	WB
5.	Actin-rabbit	ab8227	abcam	WB
6.	Gapdh-rabbit	2118	CST	WB
7.	Flotillin-2-rabbit	F1805	Sigma	WB
8.	Tsg101-rabbit	ab83	abcam	WB
9.	Smoothened-rabbit	ab236465	abcam	WB
10.	MCl-1-rabbit	4572	CST	WB
11.	BCl-xL-rabbit	2762	CST	WB
12.	Patched-rabbit	Ab53715	Abcam	WB, IHC
13.	HRP-donkey anti-rabbit, secondary	111-035-006	Jackson ImmunoResearch	WB, IHC
14.	HRP-donkey anti-mouse, secondary	715-035-150	Jackson ImmunoResearch	WB
15.	Acetyl alpha tubulin, mouse	12152	CST	ImmunoFluorescence
16.	ARL13b, rabbit	17711-1-AP	Proteintech	ImmunoFluorescence
17.	Pericentrin-rabbit (polyclonal)	ab4448	Abcam	ImmunoFluorescence
18.	Alexa Fluor 568 donkey anti-rabbit IgG (H+L)	A10042	Invitrogen	ImmunoFluorescence
19.	Alexa Fluor 488 donkey anti-mouse IgG (H+L)	A32723	Invitrogen	ImmunoFluorescence

## Supplementary information

Supplementary Figures

Supplementary Tables
