# Supplementary material for: Exovesicular-Shh confers Imatinib resistance by upregulating Bcl2 expression in chronic myeloid leukemia with variant chromosomes
Source: Cell Death Dis. 2021 Mar 11;12(3):259. doi: 10.1038/s41419-021-03542-w (PMC7952724; doi:10.1038/s41419-021-03542-w)
Supplement: Supplementary file 1 — Supplementary Figures [file 41419_2021_3542_MOESM1_ESM.pdf]

Supplementary Figures:

Supplementary Figure 1:

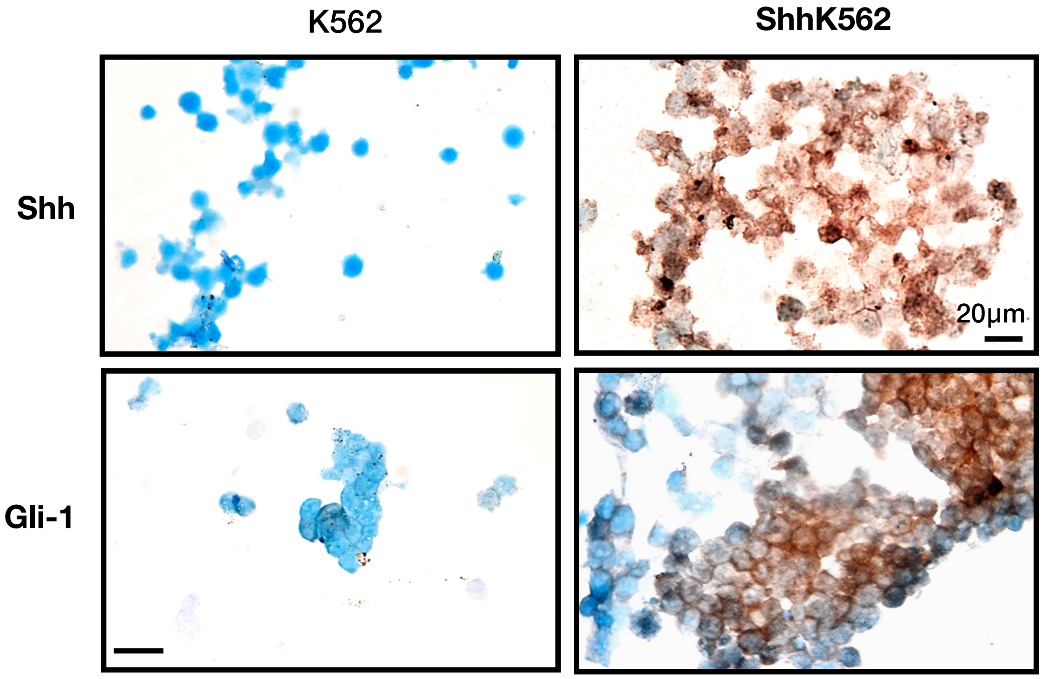

Supplementary Figure 1: Shh-expression in K562 cells leads to up-regulation of endogenous Gli-1. Immunohistochemistry on K562 (left panels) and Shh-K562 (right panels); Shh staining in top-panels and Gli-1 staining in bottom panels. Blue, Hematoxylin, Size bar= 20µm.

Supplementary Figure 2:

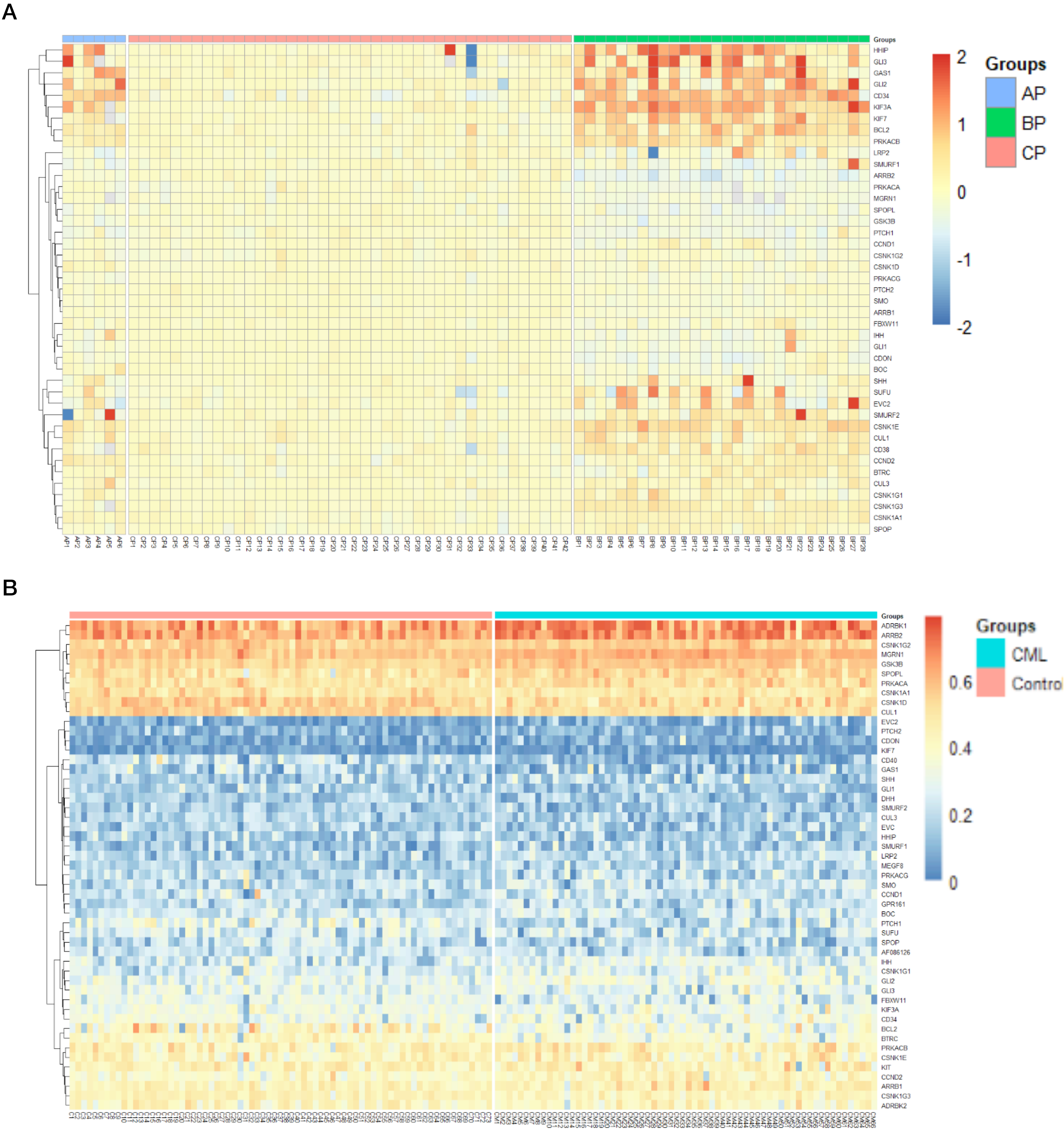

Supplementary Figure 2: The heatmap representing expression profiles of genes involved in Hedgehog pathway for accelerated phase (AP), Blast Phase (BP) and Chronic Phase (CP) conditions. A. The expression profile of samples enriched in the Hedgehog pathway where AP, CP and BP represent Accelerated Phase, Chronic Phase and Blast Crisis respectively (GSE4170). There is no observable change in the expression pattern of *SHH* gene in most samples from AP, CP or BP; where as *GLI3, Gli2*, *GAS1*, *HHIP*, *CD34* and *BCL2* has shown up regulation in their expression pattern in AP and BC samples compared to CP samples. B. Expression profile of Hedgehog pathway in Chronic Phase and healthy controls shows no difference in the pathway gene expression profile (GSE13204).

Supplementary Figure 3:

A

**Treatment**	**IC_{50} ± S.D. (nM) for 1000 cells**	
	**K562**	**Shh-K562**
**Imatinib mesylate**	270 ± 20 nM	593.33 ± 40.41 nM
	**IC_{50} ± S.D. (nM) for 1*10^6 cells**	
	710 ± 79.37 nM	1366.67 ± 77.67 nM

B

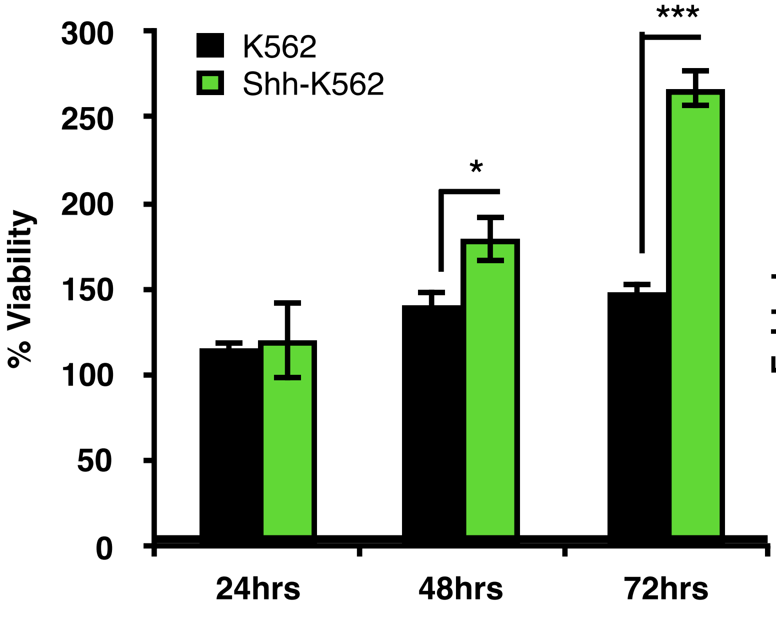

C

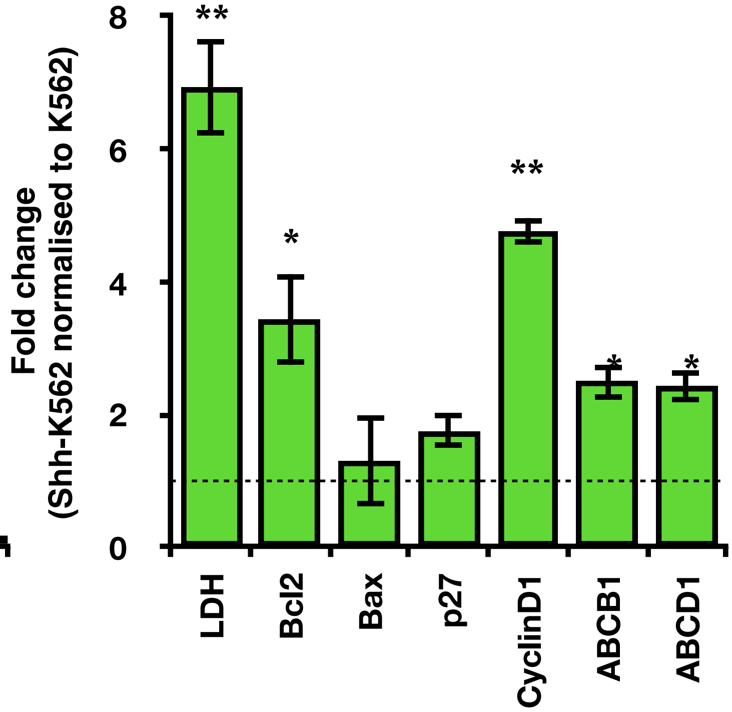

Supplementary Figure 3: Shh signalling cells are resistant to Imatinib and demonstrate better cell proliferation ability. A) Table representing Imatinib mesylate 50% inhibitory concentration using 1000 cells and million cells at 72 hours. B) Bar-graph representing percentage viability of Shh-K562 cells (green) and K562 cells (black) measured after plating cells in growth medium at indicated times. C) Bar-graph representing fold change in expression levels of genes indicated on the X-axis in Shh-K562 cells normalised to expression of these genes in K562 cells. Bar graphs represent mean ± SD from three biological repeats. Pvalues, *<0.05, **<0.01 and ***<0.001. Black dotted line represents normalising control levels.

Supplementary Figure 4:

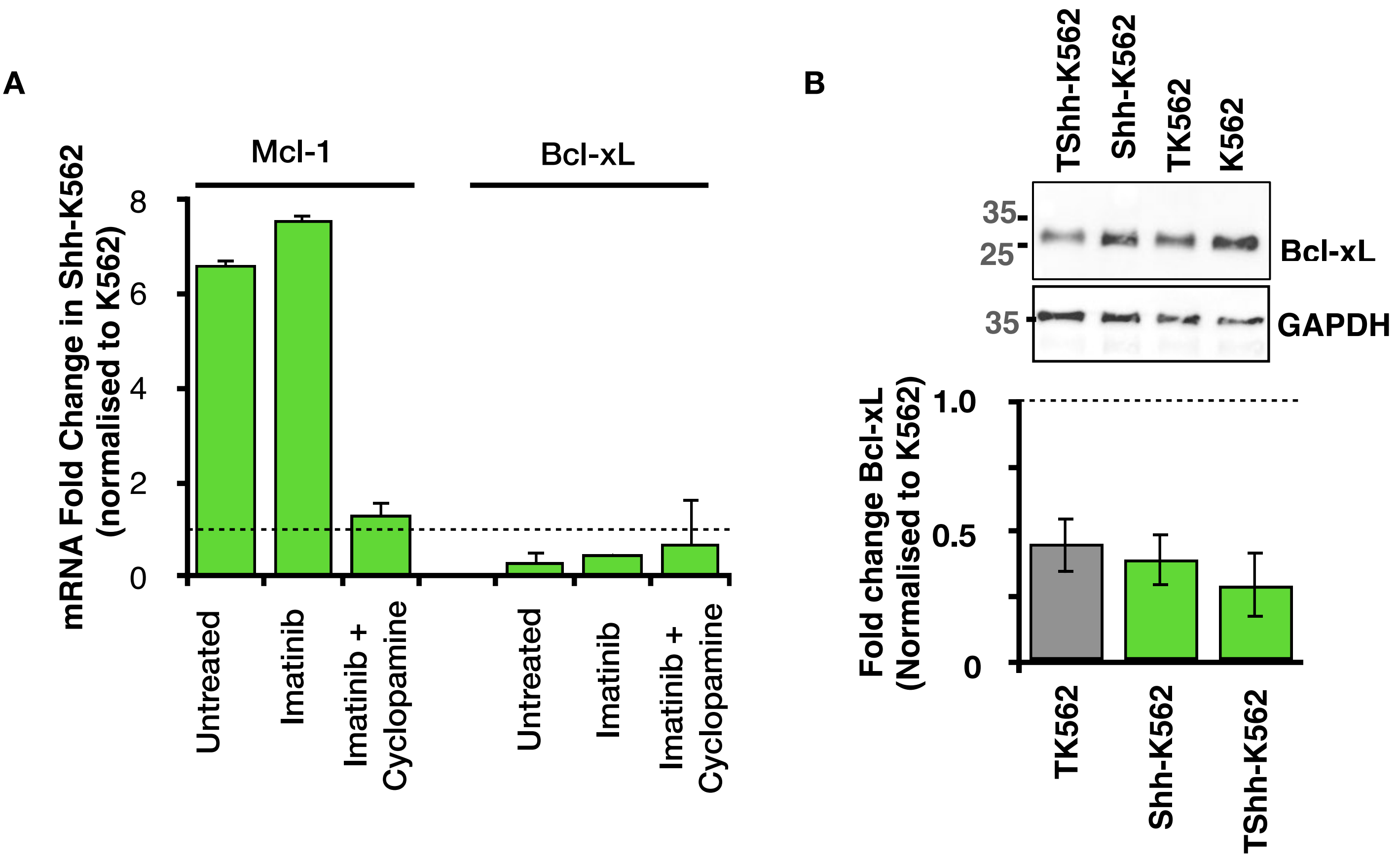

Supplementary Figure 4: Shh-signalling can regulate Mcl-1 levels but not Bcl-xL. A) Bar-graph representing RT-PCR based evaluation of Mcl-1 and Bcl-xL transcripts in untreated, Imatinib treated (0.5μM) and Imatinib (0.5μM)+Cyclopamine (25nM) treated cells at 72 hours normalised to expression in K562-cells. C) Western blot representing Bcl-xL levels in different Imatinib treated K562 (TK562), Shh-K562 and Imatinib treated Shh-K562 (TShh-K562) cells. Gapdh is used as loading control. Bar-graph representing densitometric evaluation of Bcl-xL protein levels normalised to K562 after respective GAPDH normalisation. P-values, *<0.05, **<0.01 and ***<0.001. Black dotted line represents normalising control levels.

Supplementary Figure 5:

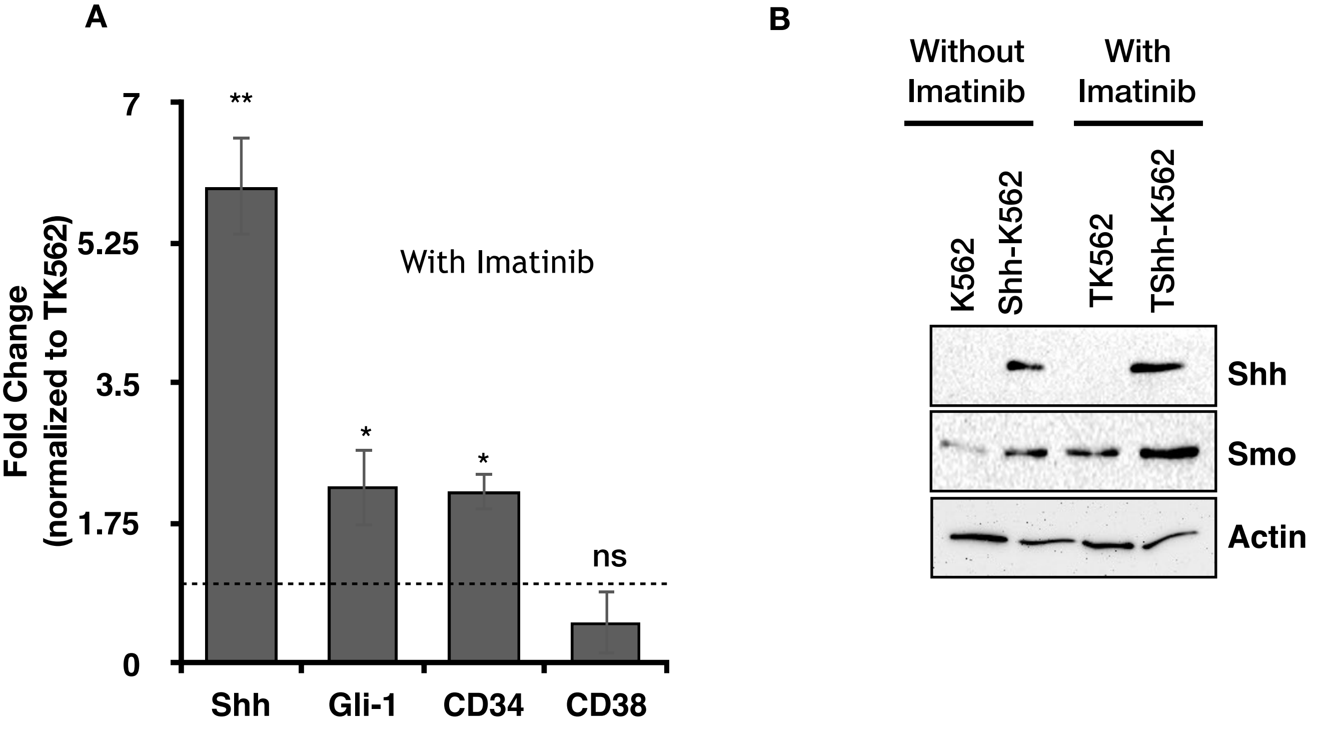

Supplementary Figure 5: Imatinib suppresses Shh expression and signalling. A) RT-PCR based evaluation of expression levels. Expression of CD34 and Gli-1 is relatively down-regulated (compare figure 2C) in presence of Imatinib. Shh and CD38 remains unaffected. P-values represent difference in expression of these genes between Imatinib treated Shh-K562 and treated K562 cells. P-values, *<0.05, **<0.01 and ns= non-significant. B) Western blot representing levels of Shh and Smo- protein in Shh-K562, Shh-K562, Imatinib treated K562 (TK562) and Imatinib treated Shh-K562 (TShh-K562) cells, 72hrs (n=2). Actin used as loading control. Black dotted line represents normalising control levels.

Supplementary Figure 6:

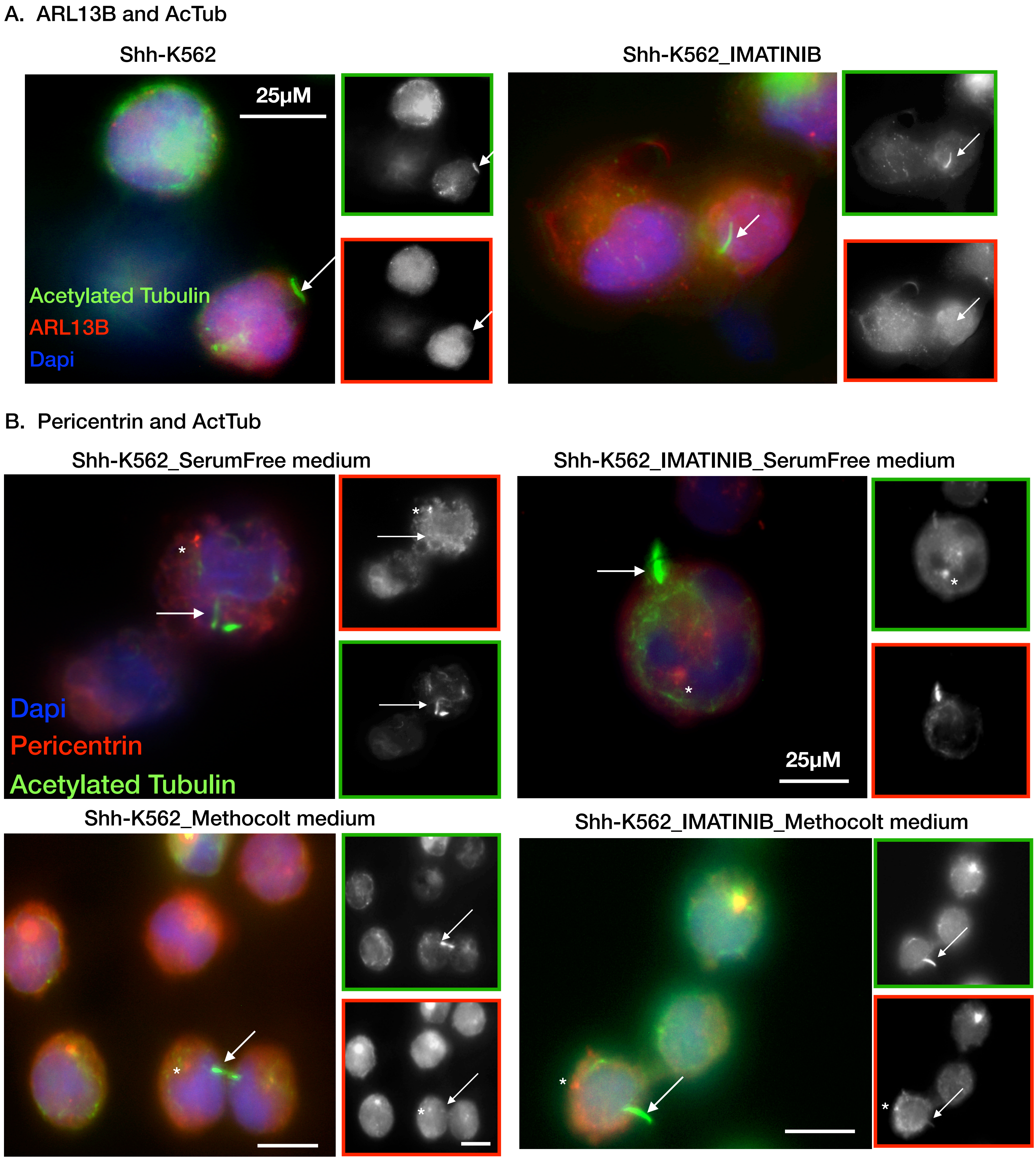

Supplementary Figure 6: Shh-K562 cells do not have detectable primary cilium. (i) Images representing Shh-K562 cells stained for ARL13b (in red, merge and inset) and Acetylated Tubulin (AcTub; green in merge and inset). (ii) Pericentrin (red in merge and inset, centriole marker) and AcTub (green in merge and inset). There was no detectable overlap between the proteins. Cells cultured in different conditions were used as indicated, for primary cilliim detection in presence or absence of Imatinib. Arrow indicates primary cilium like projection. Insets represent the red and the green channels individually. Asterisk (*) indicate location of Pericentrin in B

Supplementary Figure 7: Shh-K562 demonstrate higher sensitivity to Cyclopamine and Bcl2-inhibitor

A

Treatment	IC_{50} ± S.D. (nM) for 1000 cells	
	**K562**	**Shh-K562**
**CYCLOPAMINE alone**	126.67 ± 4.04 nM	71.67 ± 12.74 nM
**CYCLOPAMINE as adjuvant**	25 ± 1 nM	25.33 ± 0.577 nM
**YC137 alone (Bcl2 Inhibitor II)**	150.33 ± 2.5 nM	104 ± 3.6 nM
**YC137 as adjuvant**	53.33 ng ± 5.77 nM	52 ± 6.24 nM

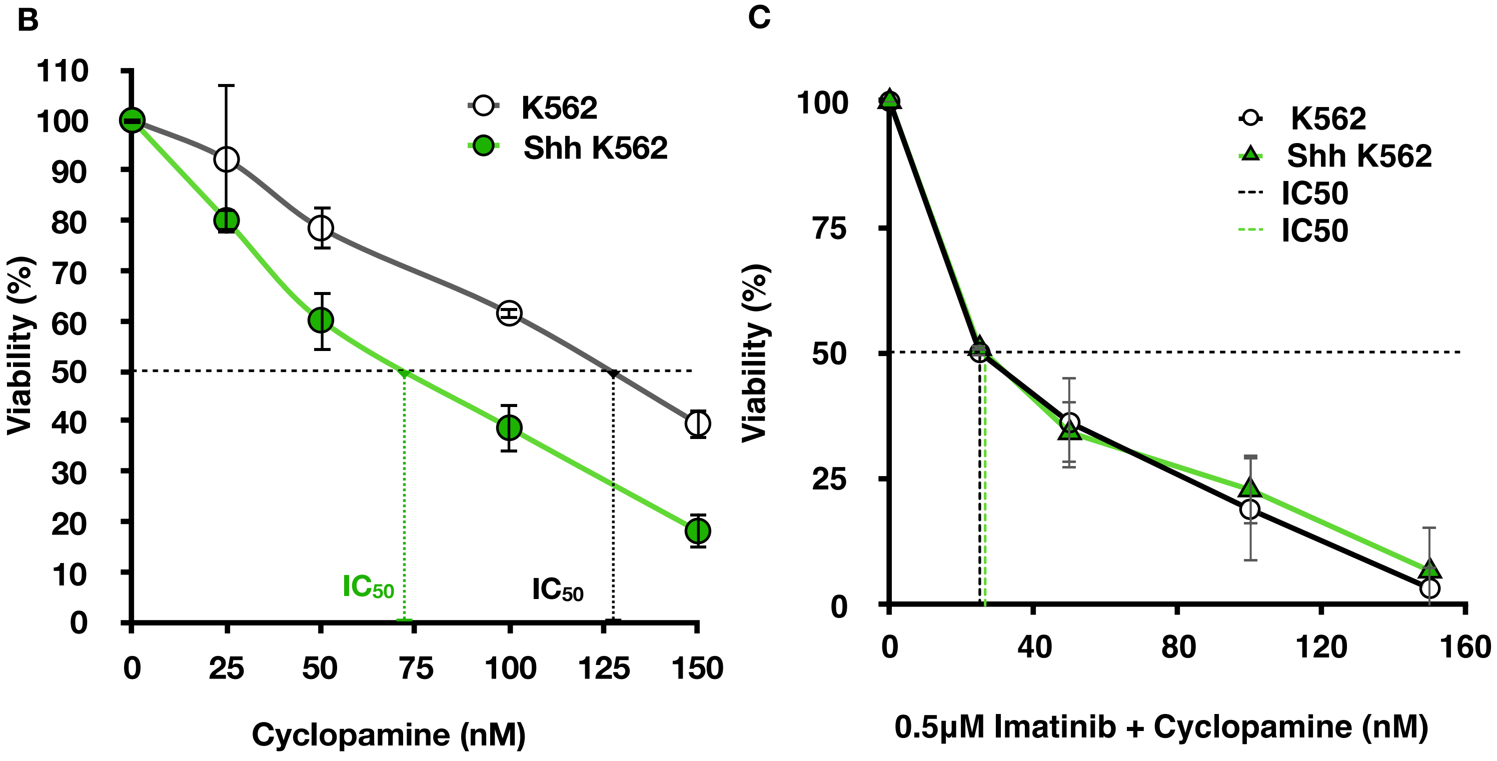

Supplementary Figure 7: Shh-K562 demonstrate higher sensitivity to Cyclopamine A) Table representing 50% inhibitory concentration of Cyclopamine, Cyclopamine as adjuvant with 0.5μM Imatinib, YC137 and YC137 as adjuvant with 0.5μM Imatinib using 1000 cells at 72 hours. B) Viability of Shh-K562 cells (green-line) and K562 cells (black line) upon treatment with different concentration of Cyclopamine (nM) for 72hrs. B) Viability of Shh-K562 cells (green-line) and K562 cells (black line) upon treatment with Imaitnib (0.5μM) along with different concentration of Cyclopamine (nM) for 72hrs. P-values represent difference in viability at specific time-point Shh-K562 and treated K562 cells. P-values, *<0.05, **<0.01 and ns= non-significant. Black dotted line represents 50% viability. All data represented comes from three biological repeats. Black dotted line, reference for 50% viability.

Supplementary Figure 8:

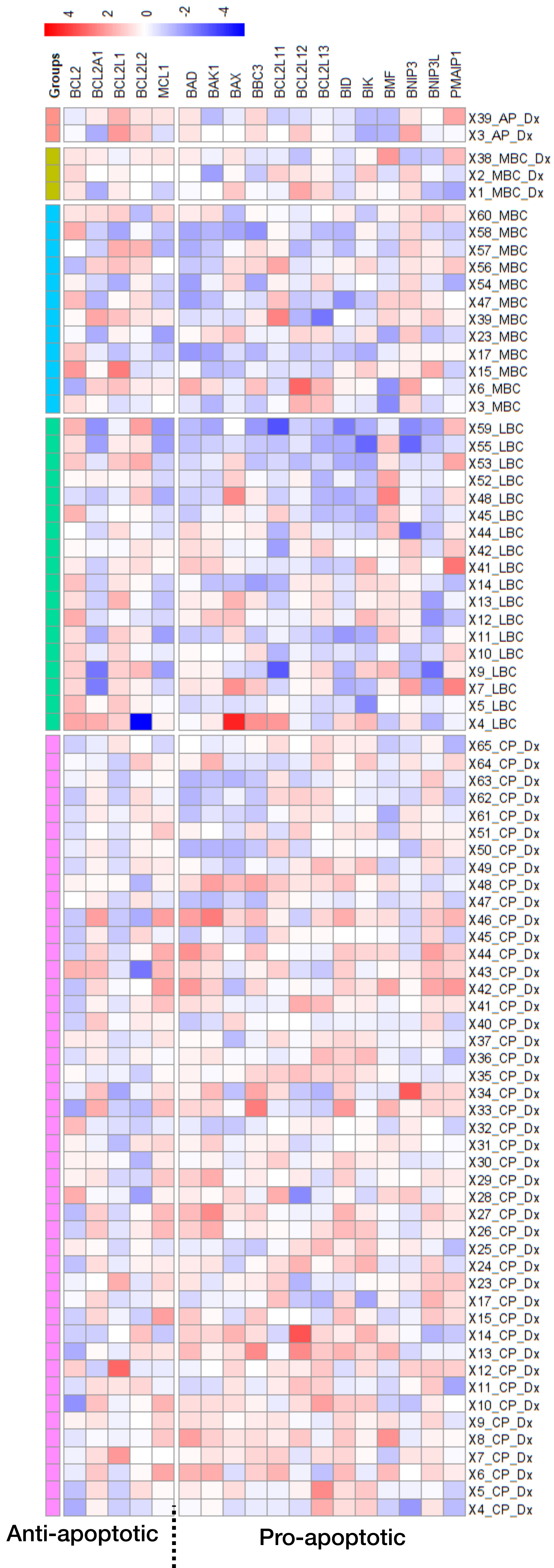

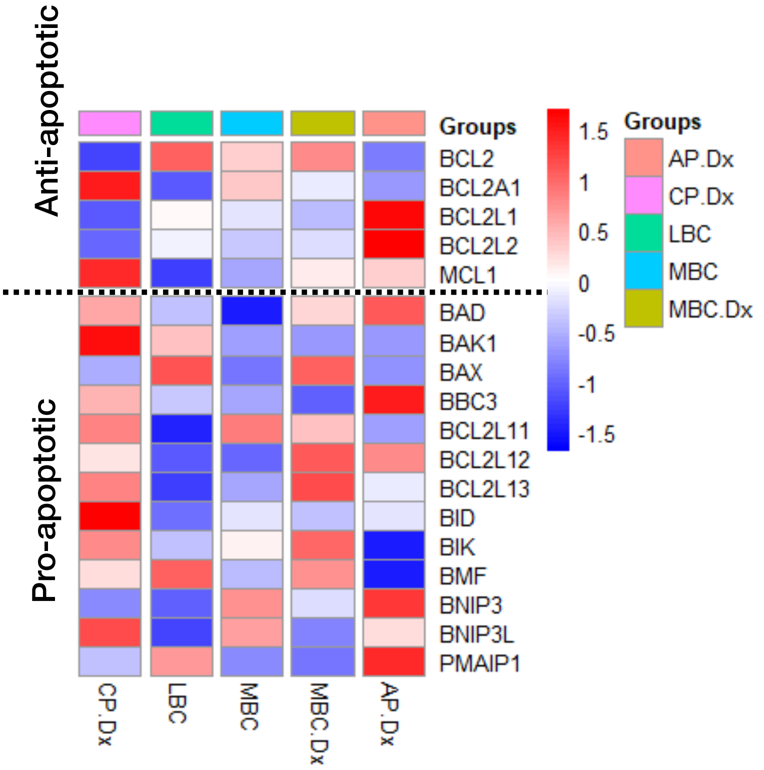

Supplementary Figure 8: Many progressive CML patients demonstrate up-regulation in expression of anti-apoptotic genes (mainly Bcl2) and down regulation of several apoptotic genes.

A. Heat-map represents the expression pattern of genes in anti-apoptotic and pro apoptotic pathways using RNA-seq dat from CML patient samples by Branford et. al. 2018. The plot represents up-regulation of anti-apoptotic gene, *BCL2,* in most progressive cases.

B. Heatmap represents mean expression of the apoptotic pathway genes in the different subtypes.

Supplementary Figure 9:

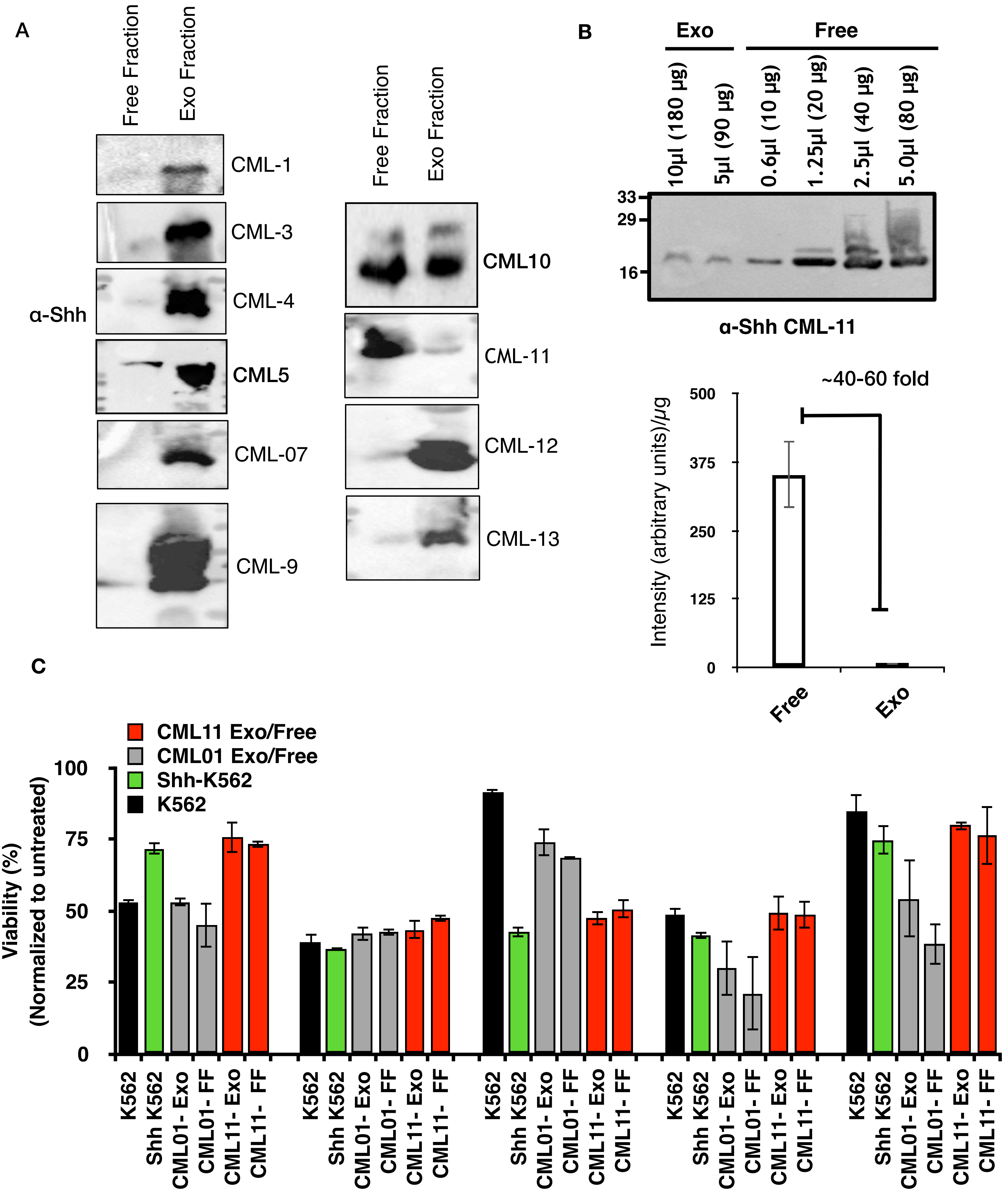

Supplementary Figure 9: Shh is present in Exo as well as Free-form in CML patient derived bone-marrow plasma. Western blot representing relative levels of Shh in Exo-fraction vs Free-fraction from each CML patient, as indicated. Equal total protein (30μg/lane) was loaded in each fraction for analysis. (B) Western blot representing presence of Shh in Exo and Free-fractions derived from CML-11 patient's bone-marrow plasma. Different concentrations of total protein (as indicated) were loaded per well to evaluate relative enrichment of Shh in each fractions. Graph represents densitometric estimation of relative protein levels normalised to total amount of protein loaded per well. C) Viability of K562 cell line treated with Exo or Free- fractions derived from patients (CML-1 and CML-11) for 12 hrs., followed by treatment with 0.5 μM Imatinib alone, 25 nM Cyclopamine alone, 50nM YC137 (Bcl2-inhibtior II) alone or in combinations (0.5 μM Imatinib + 25 nM Cyclopamine ; 0.5 μM Imatinib + 50 nM Bcl2 inhibitor), for 72hrs. Bar graph represents mean ± SD from two technical repeats.

Supplementary Figure 10: Protein content of Exo and Free-fractions

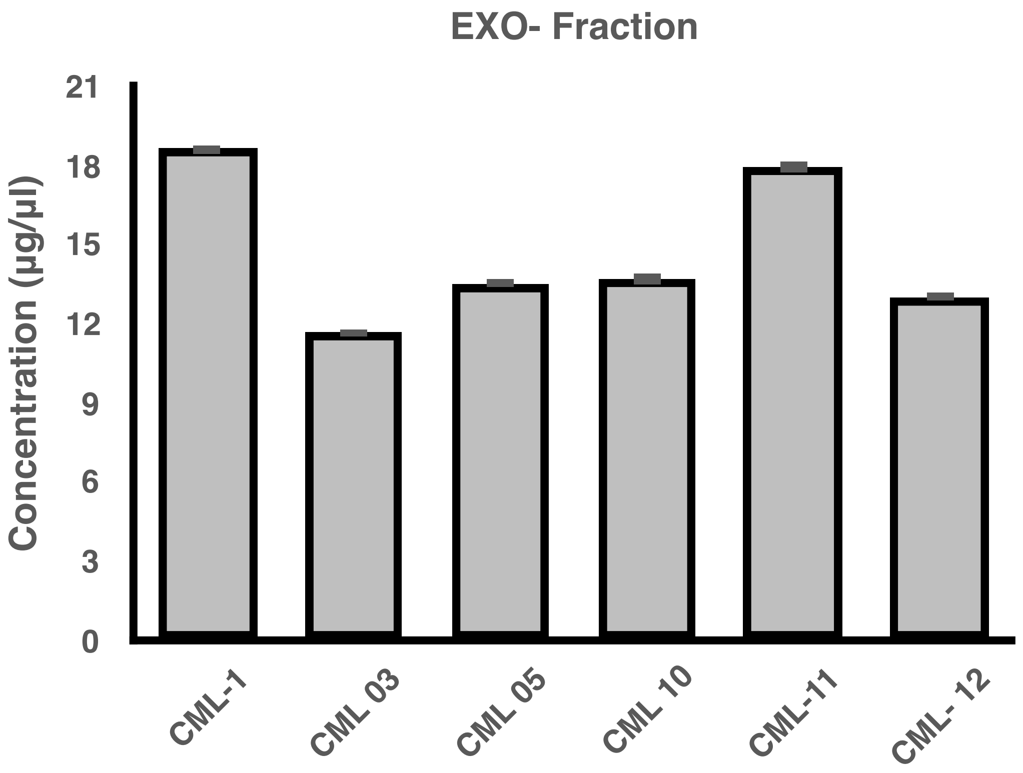

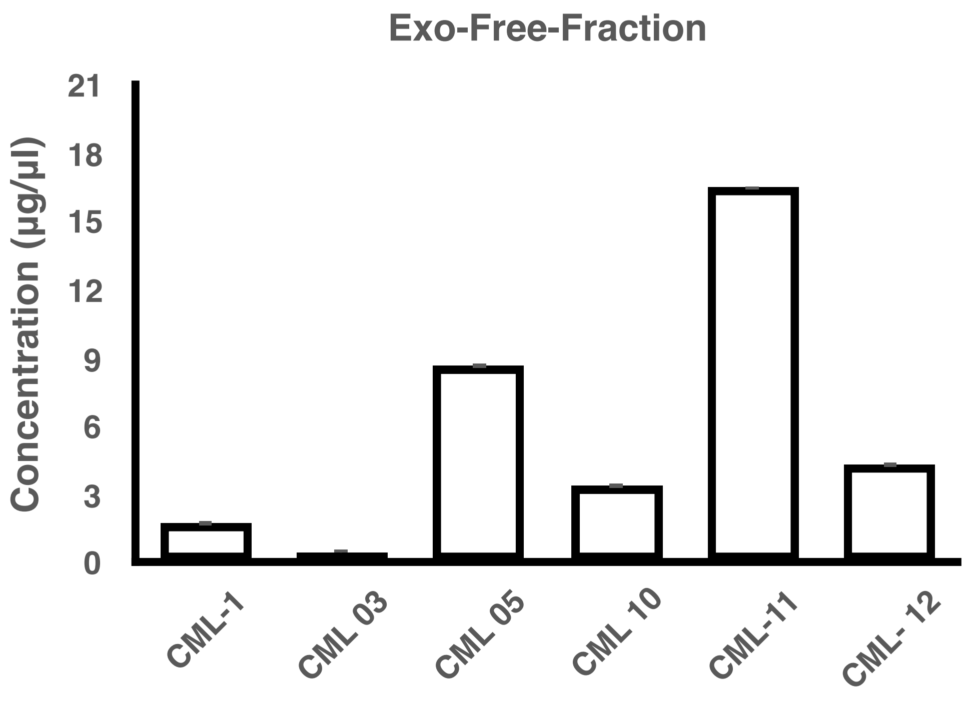

Supplementary Figure 10: Protein content of Exo and Free-fractions derived from CML patients bone-marrow aspirates. Bar-graph representing total protein content of CML patients bone-marrow plasma derived Exo and Free-Fraction as estimated by Bradford method. Mean ± SD, derived from three technical repeats.
